# Template-directed ligation of recognition-encoded melamine oligomers

**DOI:** 10.1039/d5sc05650k

**Published:** 2025-08-13

**Authors:** Laura A. Beale, Joseph T. Smith, Cecilia J. Anderson, Oliver N. Evans, Christopher A. Hunter

**Affiliations:** a Yusuf Hamied Department of Chemistry, University of Cambridge Lensfield Road Cambridge CB2 1EW UK herchelsmith.orgchem@ch.cam.ac.uk

## Abstract

The templated ligation of DNA oligomers allows quantitative coupling reactions at very low concentrations and with very high selectivity. Recognition-encoded melamine oligomers (REMO) also form sequence-selective duplexes, and it should therefore be possible to ligate them in the same way. REMO duplex formation is based on H-bonding interactions between phosphine oxide (A) and 4-nitrophenol (D) recognition units, so two DDD oligomers could be ligated using an AAAOAAA template (where O represents a blank recognition unit). Two different types of chemistry were investigated: S_N_Ar coupling of a piperazine with a dichlorotriazine, and CuAAC coupling of an azide and an alkyne. Quantitative templated ligation was observed in the presence of competing reagents, whereas statistical mixtures of different products were obtained in the absence of template. The effective molarities for the intramolecular reaction between the two substrates bound to the template are 5 mM for the S_N_Ar coupling and 3 mM for the CuAAC coupling, which means that template-directed REMO ligations at micromolar concentrations give quantitative yields with very high selectivity in the presence of large amounts of competing reactants.

## Abstract 2

Ligation of two short REMO strands on a longer sequence-complementary template gives quantitative coupling reactions at very low concentrations and with very high selectivity using either S_N_Ar or CuAAC chemistry.

## Graphical abstract

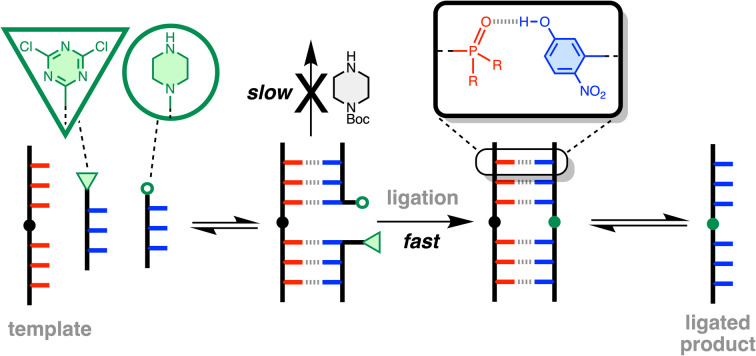

## Introduction

**Fig. 1:**
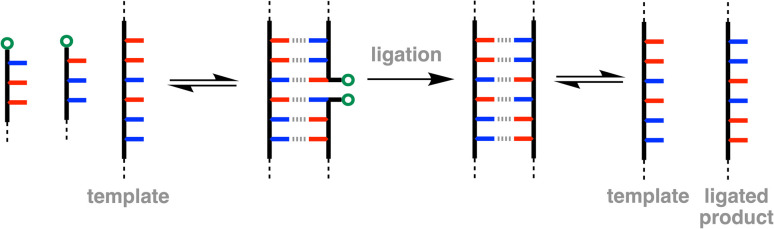
Ligation of two oligonucleotides involves binding to a template strand *via* non-covalent interactions between complementary bases (red and blue), followed by a covalent coupling reaction to link the two end groups (green circles) and generate the ligated product.

The manipulation of DNA oligomers by cutting and pasting fragments of different sequences has led to a wide range of applications in molecular biology.^1,2^ Some of the essential tools are ligase enzymes, which are used to selectively form a phosphodiester between the ends of two different DNA oligomers that are aligned by binding to a complementary template strand (Fig. 1). Sticky end ligation has been used to assemble multiple fragments into synthetic plasmids for use in biology,^3–5^ and to build complex DNA architectures for use in nanotechnology.^6–9^ Non-enzymatic ligation of oligonucleotides has also been demonstrated, and Vernier templating has been used to generate DNA duplexes with lengths comparable to a gene.^10,11^ Copper-catalysed azide–alkyne cycloaddition (CuAAC) has been used to ligate oligonucleotides equipped with terminal alkyne and azide groups to yield synthetic genes, ribozymes and catenanes, where some of the phosphodiester groups are replaced by triazoles.^12–15^

A major advantage of template-directed ligation is that the reactions can be carried out at much lower concentrations than conventional bimolecular chemistry. The rate of ligation is determined by the effective molarity (EM) of the intramolecular reaction that joins the two chains ends bound to the template, and the EM can be orders of magnitude higher than the solution phase concentration of the reactants. As a result, rapid ligation reactions that proceed in high yield and selectivity can be obtained without the need for enzyme catalysis, because the rates of competing intermolecular reactions are much lower. Here we describe the development of analogous ligation methods for template-directed assembly of synthetic polymers from shorter fragments.

**Fig. 2:**
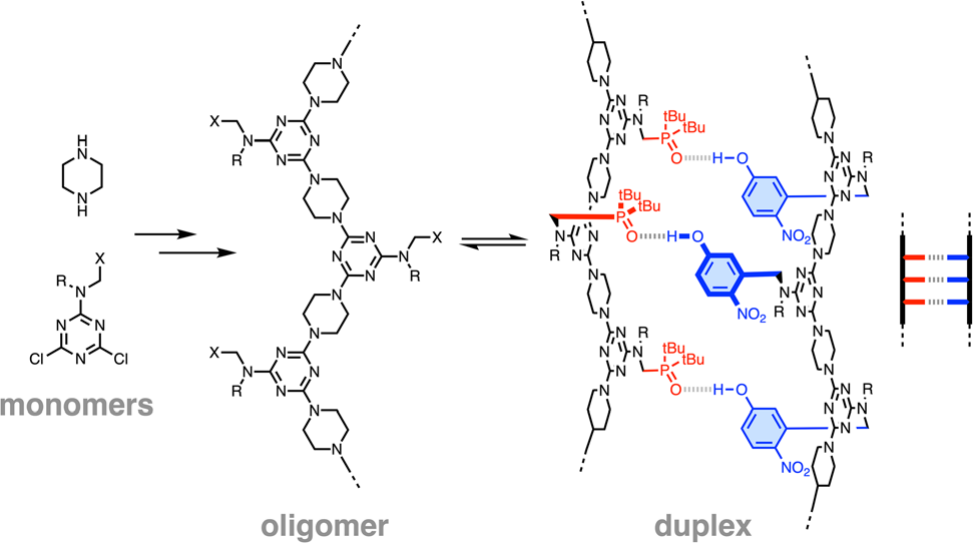
Recognition-encoded melamine oligomers (REMO) are synthesized using sequential S_N_Ar reactions of piperazine and dichlorotriazines equipped with recognition groups (X) and solubilizing groups (R). Complementary oligomers form duplexes *via* H-bond base-pairing interactions between 4-nitrophenol (D) and phosphine oxide (A) side-chains (a cartoon representation of the AAA·DDD duplex is shown).

We recently reported an automated solid-phase synthesis method for the preparation of recognition-encoded melamine oligomers (REMO), which form sequence-selective duplexes in the same way as nucleic acids (Fig. 2).^16–19^ However, the length of oligomers that are accessible by solid-phase synthesis is ultimately limited, and the ligation strategies used in molecular biology offer an attractive approach to building long REMO polymers from shorter oligomeric fragments. REMO are composed of an alternating 1,3,5-triazine-piperazine backbone, and the sequence is defined by side-chains equipped with complementary recognition units, phosphine oxide (A) or 4-nitrophenol (D) (Fig. 2). H-Bonding interactions between the recognition units lead to the assembly of duplexes from oligomers with complementary sequences, and we have shown that these base-pairing interactions can also be used for template-directed replication of short oligomer sequences.^20^ Here we report the development of two different strategies for the selective ligation of REMO using template-directed coupling reactions based on S_N_Ar and CuAAC chemistry.

## Approach

**Fig. 3:**
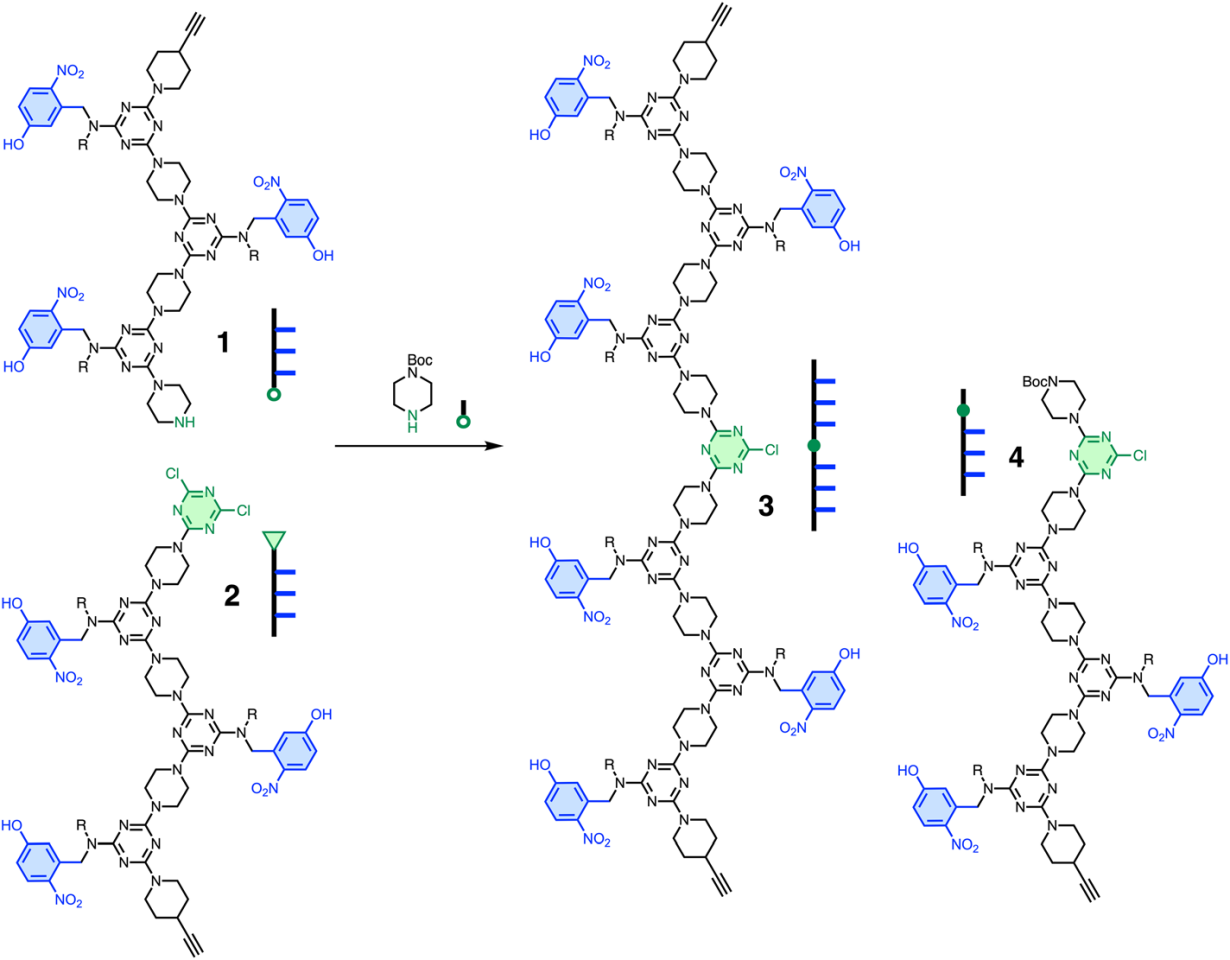
S_N_Ar ligation of two DDD oligomers, 1 and 2, in the presence of *N*-Boc-piperazine gives two different products, 3 and 4. Cartoon representations of the oligomers are shown. R = 2-ethylhexyl.

**Fig. 4:**
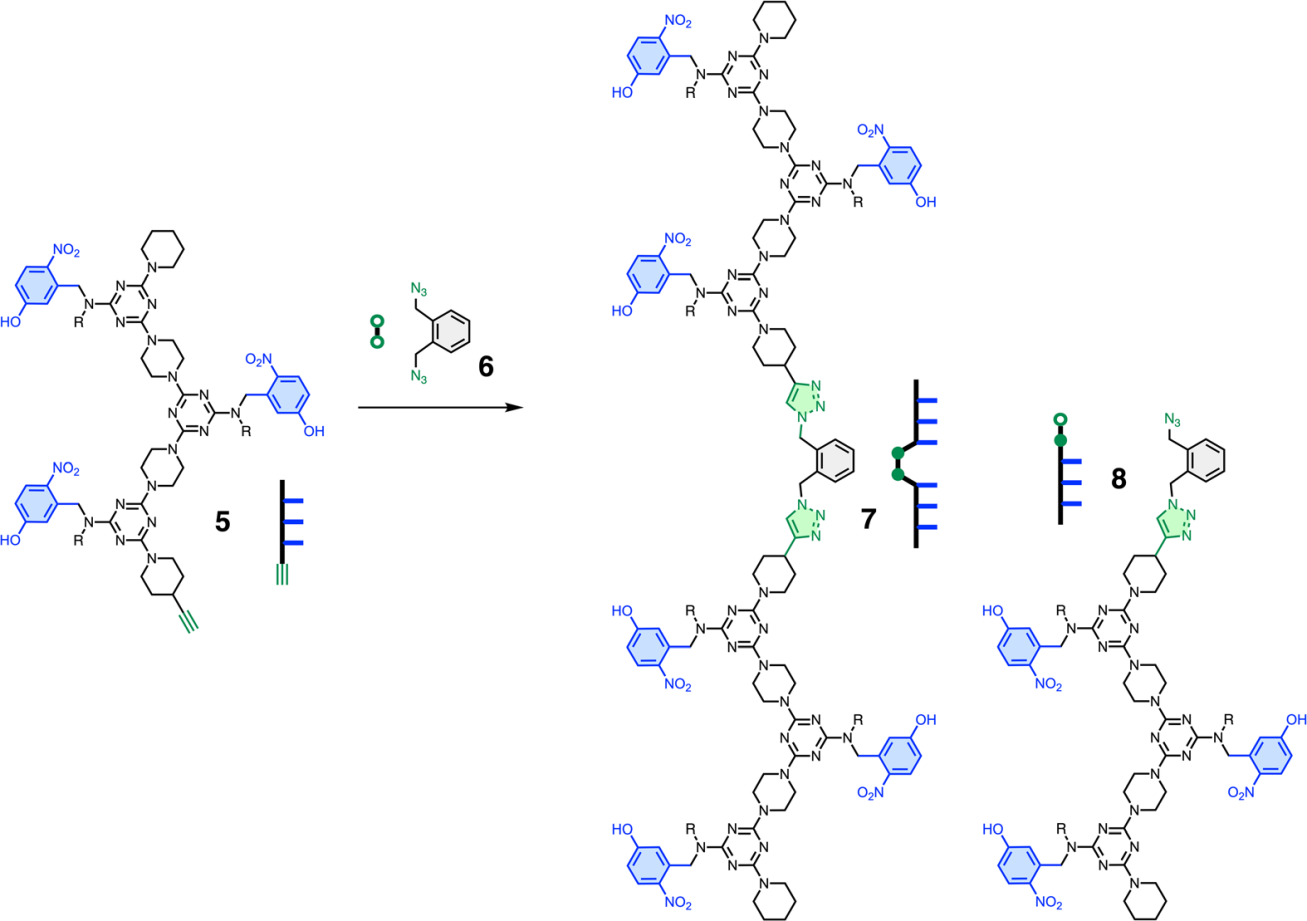
CuAAC ligation of DDD oligomer 5 with diazide 6 gives two different products, 7 and 8. Cartoon representations of the oligomers are shown. R = 2-ethylhexyl.

**Fig. 5:**
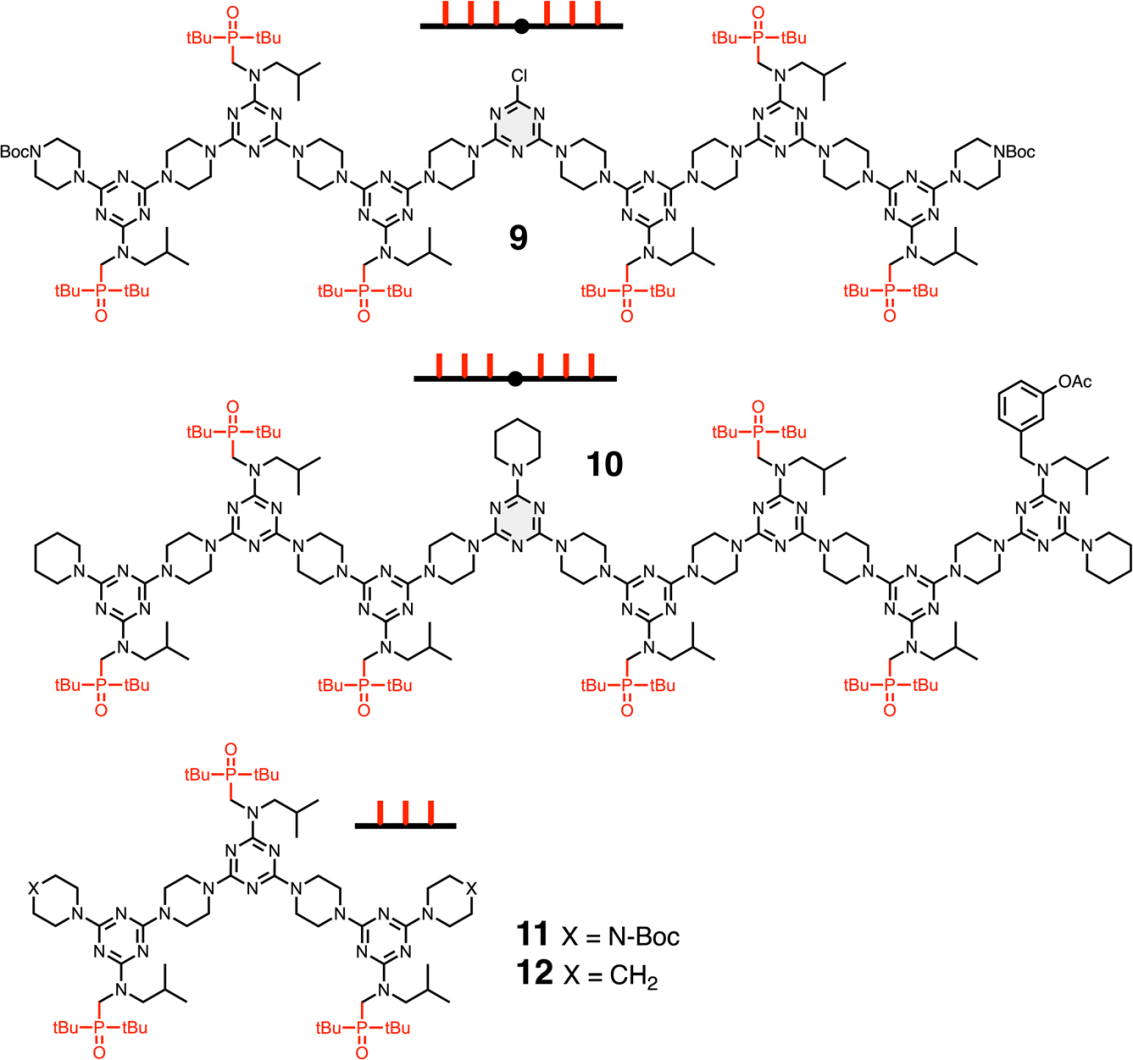
AAAOAAA oligomers 9 and 10 used to template ligation reactions, and AAA oligomers 11 and 12 used for control experiments. Cartoon representations of the oligomers are shown.

Fig. 3 shows S_N_Ar coupling of REMO 1, a DDD oligomer equipped with a terminal piperazine (nucleophilic nitrogen highlighted in green), with REMO 2, a DDD oligomer equipped with a terminal dichlorotriazine (green). Fig. 4 shows CuAAC coupling of a diazide with two copies of REMO 5, a DDD oligomer equipped with a terminal alkyne (green). The REMO substrates in Fig. 3 and 4 all contain three H-bond donor recognition sites, so oligomers 9 and 10 in Fig. 5, which each contain six H-bond acceptor recognition sites, were investigated as templates for the ligation reactions. The templates each contain two AAA sequences separated by a blank site (O), so that they are complementary in length to the ligated products 3 and 7. Although the blank site in template 9 is a monochlorotriazine, which might lead to competing S_N_Ar reactions, dichlorotriazines are orders of magnitude more reactive, and no reaction with the monochlorotriazine was observed under the reaction conditions used here. The AAA oligomers, 11 and 12, in Fig. 5 were used as control compounds that can bind the DDD substrates but do not have enough recognition sites to template ligation reactions.

Fig. 3 and 4 illustrate competition experiments that can be used to establish the magnitude of any template effects in these systems. In the S_N_Ar chemistry in Fig. 3, *N*-Boc-piperazine was used an intermolecular competitor for reaction with the dichlorotriazine oligomer 2. The relative yields of compounds 3 and 4 in the presence of varying amounts of *N*-Boc-piperazine can therefore be used to establish the EM for the intramolecular ligation reaction on the template. In the CuAAC chemistry in Fig. 4, addition of excess of diazide 6 was used an intermolecular competitor for the ligation reaction, and the relative yields of compounds 7 and 8 can be used to establish the EM for the intramolecular reaction on the template.

## Results and discussion

Sequential S_N_Ar reactions were used to synthesise the required REMO shown in Fig. 3–5 (1, 2, 5, and 9–12) according to procedures previously described (see SI for details).^16,17^

**Fig. 6:**
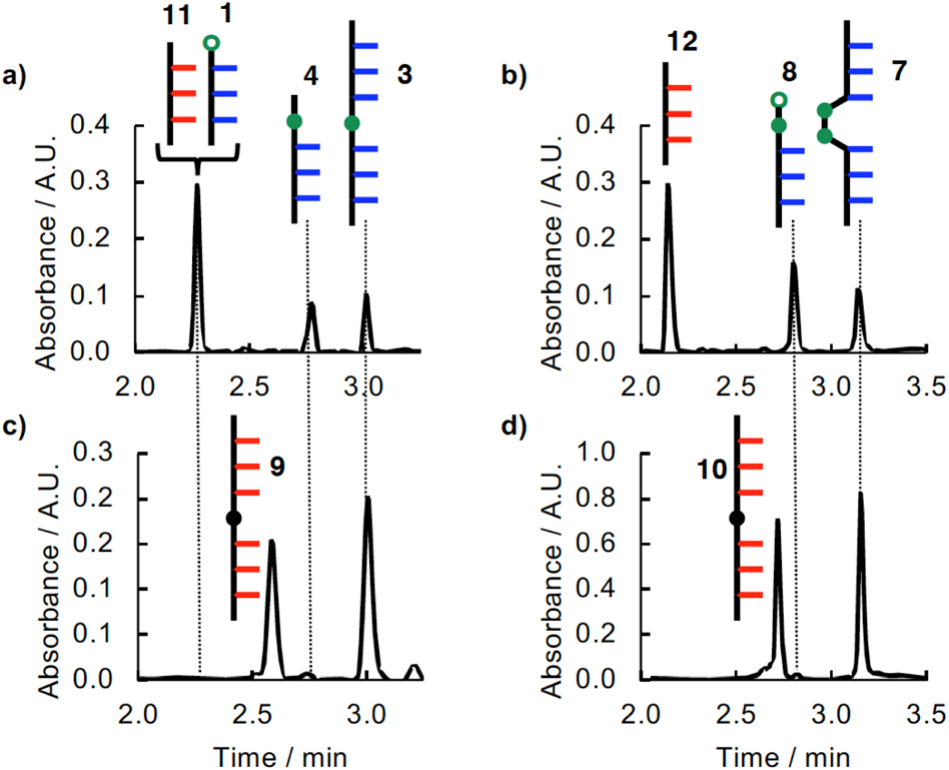
(a) UPLC traces of the crude product mixture obtained after reaction of a 1 : 1 : 1 mixture of 1, 2 and *N*-Boc-piperazine (0.1 mM) in the presence of the AAA oligomer 11 (0.2 mM) and DIPEA (1 mM) for 1 day at room temperature in DCM. (b) UPLC traces of the crude product mixture obtained after reaction of a 1 : 1 mixture of 5 and 6 (0.1 mM) in the presence of the AAA oligomer 12 (0.1 mM) and Cu^I^TBTA (0.5 mM) for 2 days at room temperature in DCM. (c) UPLC traces of the crude product mixture obtained after reaction of a 1 : 1 : 1 mixture of 1, 2 and *N*-Boc-piperazine (0.1 mM) in the presence of the AAAOAAA template 9 (0.1 mM) and DIPEA (1 mM) for 2 hours at room temperature in DCM. (d) UPLC traces of the crude product mixture obtained after reaction of a 1 : 1 mixture of 5 and 6 (0.1 mM) in the presence of the AAAOAAA template 10 (0.1 mM) and Cu^I^TBTA (0.5 mM) for 2 days at room temperature in DCM. UPLC conditions: C4 column at 40 °C (254 nm) using water + 0.1% formic acid (A) and THF + 0.1% formic acid (B); gradient of 0–4 min 30–100% B + 2 min 100% B.

The S_N_Ar coupling reaction shown in Fig. 3 was investigated using a 1 : 1 : 1 mixture of 0.1 mM 1, 2 and *N*-Boc-piperazine in dichloromethane solution in the presence of an excess of *N*,*N*-diisopropylethylamine (DIPEA). Fig. 6a shows the UPLC trace of the crude product mixture obtained after one day when the reaction was carried out in the presence of the AAA oligomer 11. Two different products, 3 and 4, were formed in similar amounts, and there was a significant amount of unreacted 1, which was observed at the same retention time as the peak due to 11. In contrast, when the reaction was carried out in the presence of the AAAOAAA ligation template 9, the competing intermolecular reaction with *N*-Boc-piperazine was completely eliminated: only the ligated product 3 was observed with no unreacted starting material 1 (Fig. 6c).

The CuAAC coupling reaction shown in Fig. 4 was investigated using a 1 : 1 mixture of 0.1 mM 5 and 6 in dichloromethane solution in the presence of Cu(i) TBTA (tris((1-benzyl-4-triazolyl)methyl)amine). Fig. 6b shows the UPLC trace of the crude product mixture obtained after two days when the reaction was carried out in the presence of the AAA oligomer 12. Two different products, 7 and 8, were formed in similar amounts. In contrast, when the reaction was carried out in the presence of the AAAOAAA ligation template 10, only the ligated product 7 was observed, and the competing intermolecular reaction with the excess of 6 was completely eliminated (Fig. 6d).

**Fig. 7:**
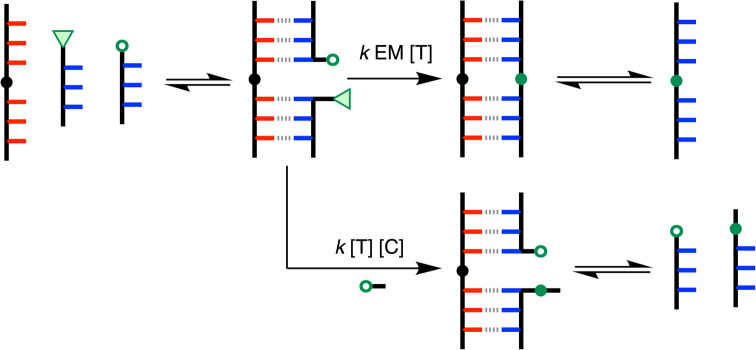
Competing intermolecular and intramolecular reaction pathways for templated ligation of two DDD oligomers in the presence of an AAAOAAA template using S_N_Ar chemistry. The second order rate constant for the coupling reaction is *k*, [*T*] is the concentration of template, [*C*] is the concentration intermolecular competitors, and EM is the effective molarity for the templated reaction.

Fig. 7 illustrates the competing reaction pathways in the templated S_N_Ar ligation reaction. The association constant for formation of the AAA·DDD duplex in dichloromethane solution is 5 × 10^6^ M^−1^,^20^ so the DDD reactants are more than 95% bound to complementary AAA sites under the reaction conditions. The relative rates of the intramolecular ligation reaction and competing intermolecular reactions are given by the ratio of the EM and the concentration of competitors, [*C*].

**Fig. 8:**
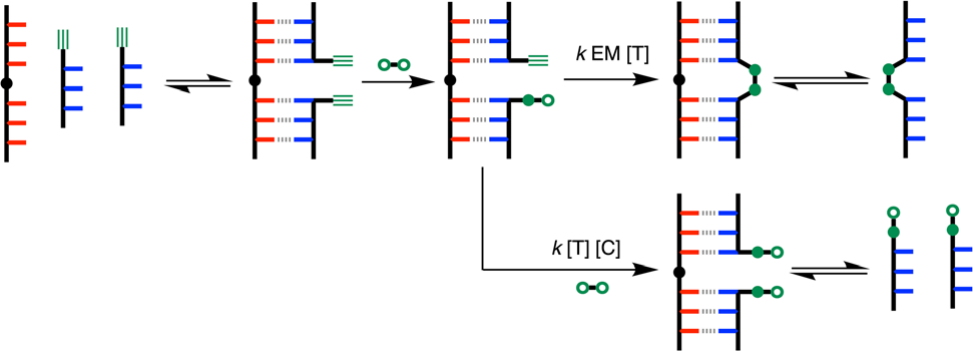
Competing intermolecular and intramolecular reaction pathways for templated ligation of two DDD oligomers in the presence of an AAAOAAA template CuAAC chemistry. The second order rate constant for the coupling reaction is *k*, [*T*] is the concentration of template, [*C*] is the concentration intermolecular competitors, and EM is the effective molarity for the templated reaction.

Fig. 8 shows the pathway for the templated CuAAC ligation. This reaction is slightly more complicated, because 5 must first react with diazide 6 to give the azide-functionalised DDD oligomer 8 (see Fig. 4). The rate-limiting step for the CuAAC reaction is formation of the copper–acetylide complex,^21^ but the key competition reaction that determines the product distribution is the competition between an intramolecular coupling of 5 with 8 bound to the template *versus* an intermolecular reaction of 5 with diazide 6. As for the S_N_Ar reaction, the relative rates of these two processes are governed by the ratio of the EM and the concentration of solution phase diazide competitor, [*C*].

The quantitative ligation reactions shown in Fig. 6 indicate that the values of EM for both the templated S_N_Ar and CuAAC reactions are much greater than 0.1 mM, which is the maximum concentration of competitors that are present at the start of the reactions.

**Fig. 9:**
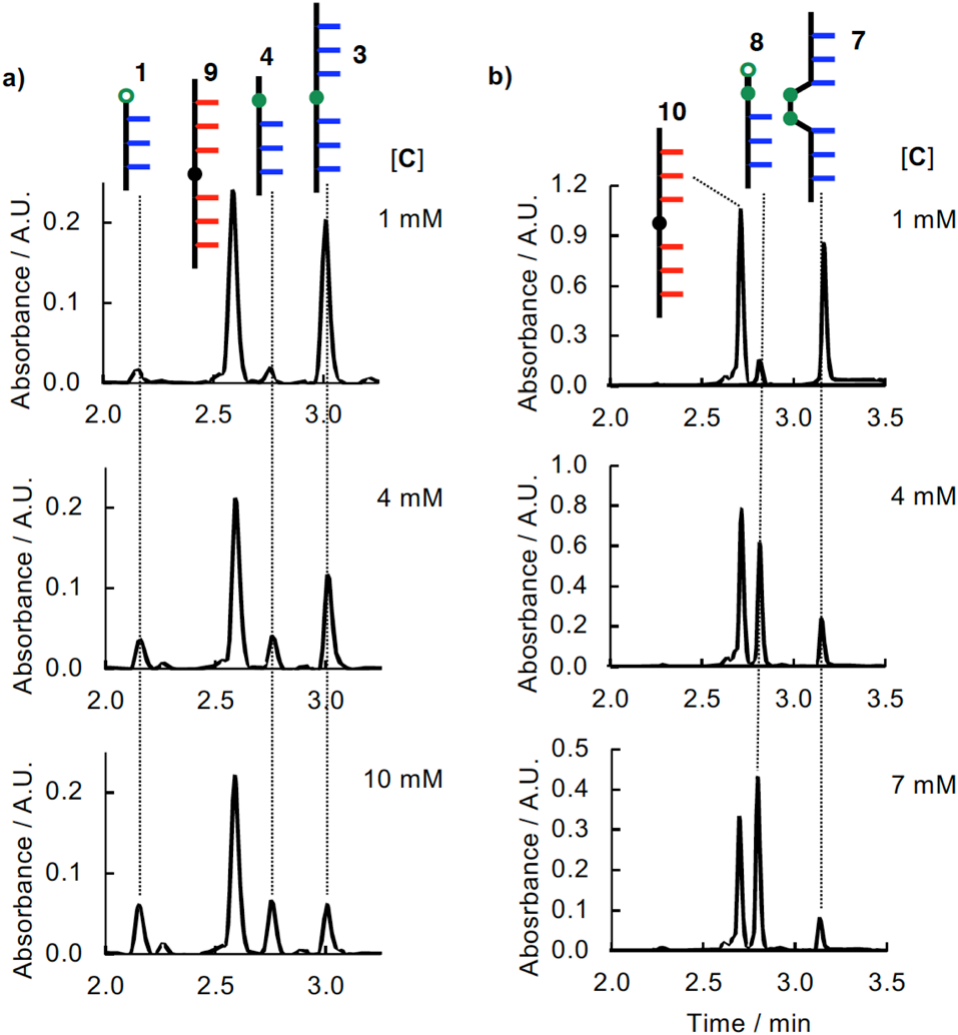
(a) UPLC traces of crude product mixtures obtained after reaction of a 1 : 1 mixture of 1 and 2 (0.1 mM) in the presence of the AAAOAAA template 9 (0.1 mM), DIPEA (1 mM) and different concentrations of *N*-Boc-piperazine ([*C*]) for 2 hours at room temperature in DCM. (b) UPLC traces of crude product mixtures obtained after reaction of 5 (0.1 mM) in the presence of the AAAOAAA template 10 (0.05 mM), Cu^I^TBTA (0.5 mM) and different concentrations of diazide 6 ([*C*]) for 2 days at room temperature in DCM. Major peaks are labelled with cartoons. UPLC conditions: C4 column at 40 °C (254 nm) using water + 0.1% formic acid (A) and THF + 0.1% formic acid (B); gradient of 0–4 min 30–100% B + 2 min 100% B.

To quantify the template effects in these systems, the ligation reactions were repeated in the presence of increasing concentrations of the intermolecular competitor, *N*-Boc-piperazine for the S_N_Ar ligation, or 6 for the CuAAC ligation. Fig. 9 shows the resulting UPLC traces of the crude product mixtures. In the presence of 10-fold excess of intermolecular competitors (1 mM), the templated ligation reactions proceed almost quantitatively to give a single product in the both the S_N_Ar reaction (Fig. 9a) and CuAAC reaction (Fig. 9b). At higher concentrations of the competing reagents, the yields of the ligated products start to drop.

Assuming that the extinction coefficients of the ligated products are approximately twice the extinction coefficients of the unligated DDD oligomers, the selectivity of the ligation reaction can be estimated by integrating the peak areas in the UPLC traces (eqn (1)).1
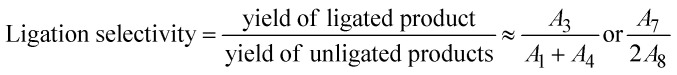
where *A*_*x*_ is the integral of the UPLC peak corresponding to compound *x*.

The ligation selectivity defined by eqn (1) is related to the relative rates of the intramolecular and intermolecular processes shown in Fig. 7 and 8. In the presence of a large excess of the competing reagent, the total concentration of intermolecular competitors [*C*] is approximated by the concentration of this reagent (eqn (2)).2
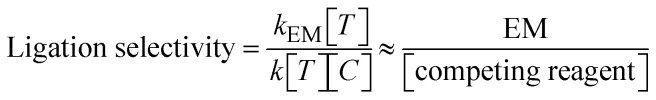


**Fig. 10:**
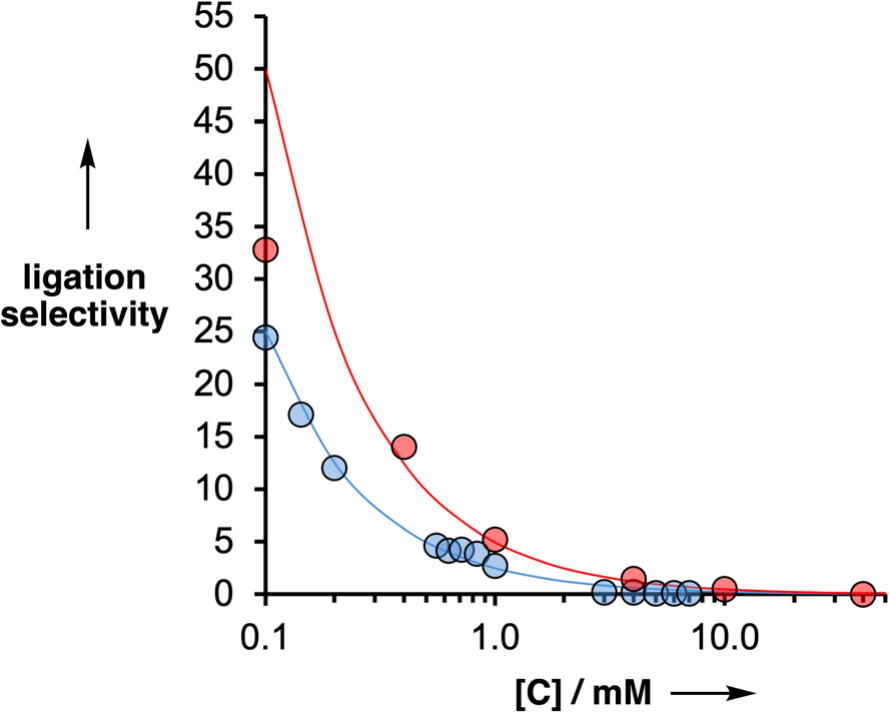
Ligation selectivity plotted as a function of the concentration of the competing reagent ([*C*]) used in S_N_Ar ligation reactions, *N*-Boc-piperazine (red), or the competing reactant used in CuAAC ligation reactions, diazide 6 (blue). The red data shows the results for the reaction of a 1 : 1 mixture of 1 and 2 (0.1 mM) in the presence of the AAAOAAA template 9 (0.1 mM). The blue data shows the results for the CuAAC reaction of 5 (0.1 mM) with 6 in the presence of the AAAOAAA template 10 (0.05 mM). The lines correspond to eqn (2) with EM = 3 mM (blue) and EM = 5 mM (red).

The product distributions were measured from the UPLC traces obtained from the templated ligation reactions carried out at different concentrations of competing reagent, and the ligation selectivities are plotted in Fig. 10 for both the S_N_Ar (red) and CuAAC (blue) reactions. The points represent the experimental data, and the lines show the best fits to eqn (2) obtained with an EM of 5 mM for the S_N_Ar ligation and 3 mM for the CuAAC reaction. Note that the red data point at 0.1 mM of the competing reactant does not fall on the line calculated for an EM of 5 mM. This datapoint was not used in the fit to eqn (2) used to determine the EM, because the yield of the templated product was almost quantitative under these conditions, and the integral of the competing product was less than 3% of the total, which leads to a much larger uncertainty in the ratio of the two integrals compared with the other datapoints.

## Conclusions

The templated ligation of oligonucleotides by ligases is used extensively in molecular biology and to construct complex DNA structures. We have shown that recognition-encoded melamine oligomers (REMO) form sequence-selective duplexes reminiscent of oligonucleotide duplexes, and this property opens the possibility of using ligation strategies to construct larger oligomers from short REMO fragments. REMO duplex formation is based on H-bonding interactions between phosphine oxide (A) and 4-nitrophenol (D) recognition units. Thus two DDD oligomers can be ligated using an AAAOAAA template (where O represents a blank site with no recognition unit). Two different types of chemistry were investigated for the ligation reaction, S_N_Ar coupling of a piperazine with a dichlorotriazine, and CuAAC coupling of an azide and an alkyne. In both cases, quantitative templated ligation of two DDD oligomers was observed in the presence of competing reagents. In contrast, statistical mixtures of different products were obtained in the absence of template. It was possible to determine the effective molarity (EM) for the intramolecular reaction between two substrates bound to a template through competition experiments in the presence of increasing concentrations of a competing reagent: the value of EM is 5 mM for the S_N_Ar coupling, and 3 mM for the CuAAC coupling. These values are very similar to the values we have measured for a range of different templated reactions using the REMO system and in line with the general observation that supramolecular effective molarities are usually of the order 10–100 mM.^16,17,20,22,23^ Ligation EMs in the mM range mean that template-directed reactions performed at micromolar concentrations give quantitative ligation with very high selectivity in the presence of large amounts of competing reactants. The two complementary ligation reactions described here provide a useful new tool for the manipulation of oligomers based on the REMO architecture.

## Author contributions

The manuscript was written through contributions of all authors.

## Conflicts of interest

There are no conflicts to declare.

## Supplementary Material

SC-016-D5SC05650K-s001

## Data Availability

All supporting data is provided in the SI. Detailed synthetic procedures, full characterization including ^1^H and ^13^C NMR spectra of all compounds. See DOI: https://doi.org/10.1039/d5sc05650k.
