# Supplementary material for: Template-directed ligation of recognition-encoded melamine oligomers
Source: Chem Sci. 2025 Aug 13;16(35):15991–6. doi: 10.1039/d5sc05650k (PMC12352616; doi:10.1039/d5sc05650k)
Supplement: SC-016-D5SC05650K-s001 [file SC-016-D5SC05650K-s001.pdf]

Supplementary Information (SI) for Chemical Science.
This journal is © The Royal Society of Chemistry 2025

Templated-Directed Ligation of Recognition-Encoded Melamine Oligomers

Laura A. Beale, Joseph T. Smith, Cecilia J. Anderson, Oliver N. Evans and Christopher A. Hunter*

Yusuf Hamied Department of Chemistry, University of Cambridge, Lensfield Road, Cambridge CB2 1EW, UK

Supplementary Information

Table of Contents

1. General Experimental Details 2
2. Compound Synthesis and Characterisation 3
Synthesis of 1 4
Synthesis of 2 7
Synthesis of 5 9
Synthesis of 6 10
Synthesis of 9 12
Synthesis of 10 15
Synthesis of 11 18
Synthesis of 12 19
3. Procedures for Templating Studies 20
References 21

1. General Experimental Details

All the reagents and materials were obtained from commercial sources and used without further purification. Dry solvents were taken from the solvent purification system Pure SolvTM by Innovative Technology, Inc.. Thin layer chromatography was carried out using Silica gel 60F on glass. Flash chromatography was carried out on an automated system (Combiflash Rf+ or Rf Lumen) using pre-packed cartridges of silica (25 µm PuriFlash Column). NMR spectra were recorded on a Bruker 400 MHz AVIII400, 400 MHz Neo Prodigy, 400 MHz QNP cryoprobe, 500 MHz TCI cryoprobe or 700 MHz TCO cryoprobe spectrometer. The residual solvent was used as the internal standard. In chloroform-*d*, ^{1}H spectra were referenced to δ 7.26 ppm and ^{13}C spectra to 77.16 ppm for the solvent signal. In DMSO-d_6, ^{1}H spectra were referenced to δ 2.50 ppm and ^{13}C spectra to 39.52 ppm for the solvent signal. All chemical shifts are quoted in ppm on the δ scale. Splitting patterns are given as follows: s (singlet), br s (broad singlet), d (doublet), t (triplet), q (quartet), sept (septet), non (nonet) and m (multiplet). ^{1}H and ^{13}C NMR were assigned using DEPT, COSY, HSQC and HMBC. Pseudo-equivalent environments are labelled with the same number and where multiple peaks arise only from rotamers, this is specified. A Waters LCT premier mass spectrometer was used to obtain the ES+ mass spectra. FT-IR spectra were measured on a PerkinElmer Spectrum One spectrometer equipped with an ATR cell. The LCMS analysis of samples was performed using Waters Acquity H-class UPLC coupled with a single quadrupole Waters SQD2. An ACQUITY UPLC BEH C4 Column, 300 Å, 1.7 µm, 2.1 mm X 50 mm was used as the UPLC column. Columns were re-equilibrated for 2 minutes after each run. Flow rate: 0.4 ml/min; Column temperature of 40 °C; Injection volume of 2 µL. The signal was monitored at 254 nm. Heated automated Solid-Phase Synthesis (SPS) was performed on a CEM Liberty Blue automated synthesiser.

2. Compound Synthesis and Characterisation

Compounds **S1-S4** were synthesised following procedures previously described in the literature.[1–3] Functionalised TentaGel S Wang resin, **S5**, was prepared according to the previously described procedure.[4]

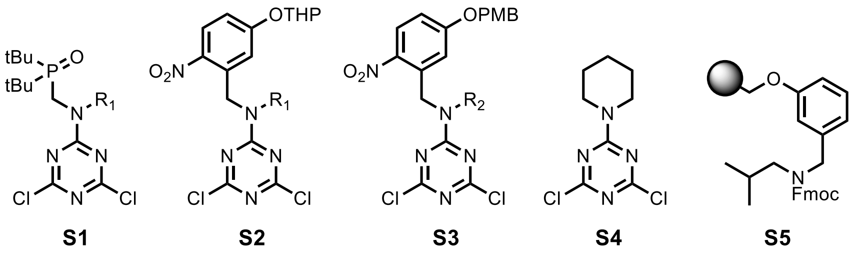

Figure S1: Structures of building blocks **S1-S5**. R_1 = isobutyl, R_2 = 2-ethylhexyl. Tentagel S Wang resin is depicted with a filled circle.

Synthesis of 1

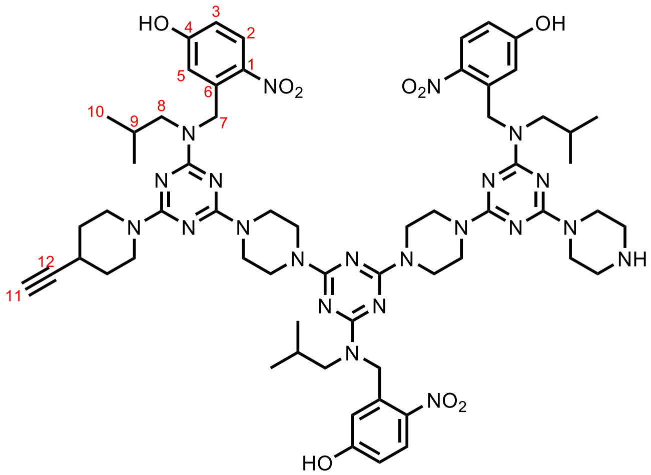

To a solution of *tert*-butyl-4-ethynylpiperidine-1-carboxylate (45.9 mg, 0.219 mmol, 1.0 eq.) in DCM (3 mL), trifluoroacetic acid (1 mL) was added and the solution was stirred for 10 minutes. The solvent was removed under a nitrogen flow and the residue azeotroped with THF (3 x 10 mL). The salt, 4-ethynylpiperidinium trifluoroacetate, was redissolved in THF (0.5 mL). To a solution of **S2** (100 mg, 0.219 mmol, 1.0 eq.) in THF (2 mL), the solution of 4-ethynylpiperidinium trifluoroacetate and DIPEA (0.4 mL, 2.2 mmol, 10 eq.) were added and the solution stirred at room temperature for 30 minutes. Piperazine (189 mg, 2.19 mmol, 10 eq.) was added and the solution diluted with THF (2 mL). The solution was heated under microwave irradiation at 60 °C for 15 minutes and then cooled to room temperature. The reaction was diluted with EtOAc (10 mL) and washed with sodium hydroxide solution (1M in water, 10 mL). The aqueous phase was extracted with EtOAc (3 x 10 mL) and the combined organic phases dried with magnesium sulfate. The solvent was evaporated *in vacuo* and the obtained residue of **S5** was used in further steps without purification.

To a solution of **S2** (100 mg, 0.219 mmol, 1.0 eq.) in THF (2 mL), *N*-Boc-piperazine (40.8 mg, 0.219 mmol, 1.0 eq.) and DIPEA (0.12 mL, 0.657 mmol, 3.0 eq.) were added and the solution stirred at room temperature for 30 minutes. Piperazine (189 mg, 2.19 mmol, 10 eq.) was added and the solution diluted with THF (2 mL). The solution was heated under microwave irradiation at 60 °C for 15 minutes and then cooled to room temperature. The reaction was diluted with EtOAc (10 mL) and washed with sodium hydroxide solution (1 M in water, 10 mL). The aqueous phase was extracted with EtOAc (3 x 10 mL) and the combined organic phases dried with magnesium sulfate. The solvent was evaporated *in vacuo* and the obtained residue of **S6** was used in further steps without purification.

To a solution of **S2** (71.1 mg, 0.159 mmol, 1.0 eq.) in THF (0.5 mL), **S5** (92.0 mg, 0.159 mmol, 1.0 eq.) and DIPEA (0.17 mL, 0.954 mmol, 6.0 eq.) were added and the solution stirred at room temperature for 20 minutes. **S6** (156 mg, 0.238 mmol, 1.5 eq.) and DIPEA (0.4 mL, 1.59 mmol, 10 eq.) were added. The solution was heated under microwave irradiation at 60 °C for 3.5 hours and then cooled to room temperature. The reaction was diluted with EtOAc (10 mL) and washed with sodium hydroxide solution (1 M in water, 10 mL). The aqueous phase was extracted with EtOAc (3 x 10 mL) and the combined organic phases dried with magnesium sulfate. The solvent was evaporated *in vacuo*. The residue was dissolved in DCM (3 mL) and trifluoroacetic acid (1 mL) was added. The reaction was stirred at room temperature for 15 minutes. Aqueous sodium hydroxide solution (3M then 1 M) was added dropwise until pH 6 was reached. The reaction was diluted with EtOAc (10 mL) and washed with water (10 mL). The aqueous phase was extracted with EtOAc (3 x 10 mL) and the combined organic phases dried with magnesium sulfate. The residues obtained were purified by silica flash chromatography (DCM/ methanol (85:15)). **1** was obtained as a yellow foam (88.4 mg, 0.0699 mmol, 44%).

^{1}H NMR (500 MHz, DMSO-d_6): δ_H = 8.02 (br, 3H, H_2), 6.74 (br, 3H, H_3), 6.51 (br, 3H, H_5), 5.01 (br, 6H, H_7), 4.18 – 3.07 (br, 26H, H_8, protons of piperazine and piperidine rings), 2.97 and 2.96 (d, J = 2.2 Hz, 1H, H_{11}, rotamers), 2.93 – 2.53 (br, 8H, protons of piperazine and piperidine rings), 2.08 (br, 3H, H_9), 1.87 – 1.14 (br, 5H, protons of piperidine ring), 0.87 (br, 18H, H_{10}).

^{13}C NMR (126 MHz, DMSO-d_6): δ_C = 165.4, 164.7, 164.6, 164.6, 164.5, 164.5, 164.4, 164.4, 164.3 (carbons of triazine rings), 163.2 (C_4), 139.0 (C_1), 138.8 (C_6), 128.2 and 128.1 (C_2), 114.2 (C_3), 113.8 (C_5), 87.0 and 86.9 (C_{12}, rotamers), 72.0 and 71.9 (C_{11}, rotamers), 54.3 and 54.1 (C_8), 48.5 (C_7), 44.2, 44.0, 43.9, 42.5, 42.3, 41.3, 41.2 (carbons of piperazine and piperidine rings), 31.0 and 30.8 (piperidine carbon, rotamers), 29.0 (piperidine carbon), 27.3, 27.2, 27.2 (C_9), 26.4 and 26.3 (piperidine carbon, rotamers), 20.2 (C_{10}).

HRMS (ES+): Calculated for $C_{61}H_{81}N_{22}O_9^+$, 1265.6551; found 1265.6559.

FT-IR (ATR): ν_{max} 3336, 2955, 1580, 1532, 1480, 1433, 1333, 1306, 1260, 1208, 998, 807

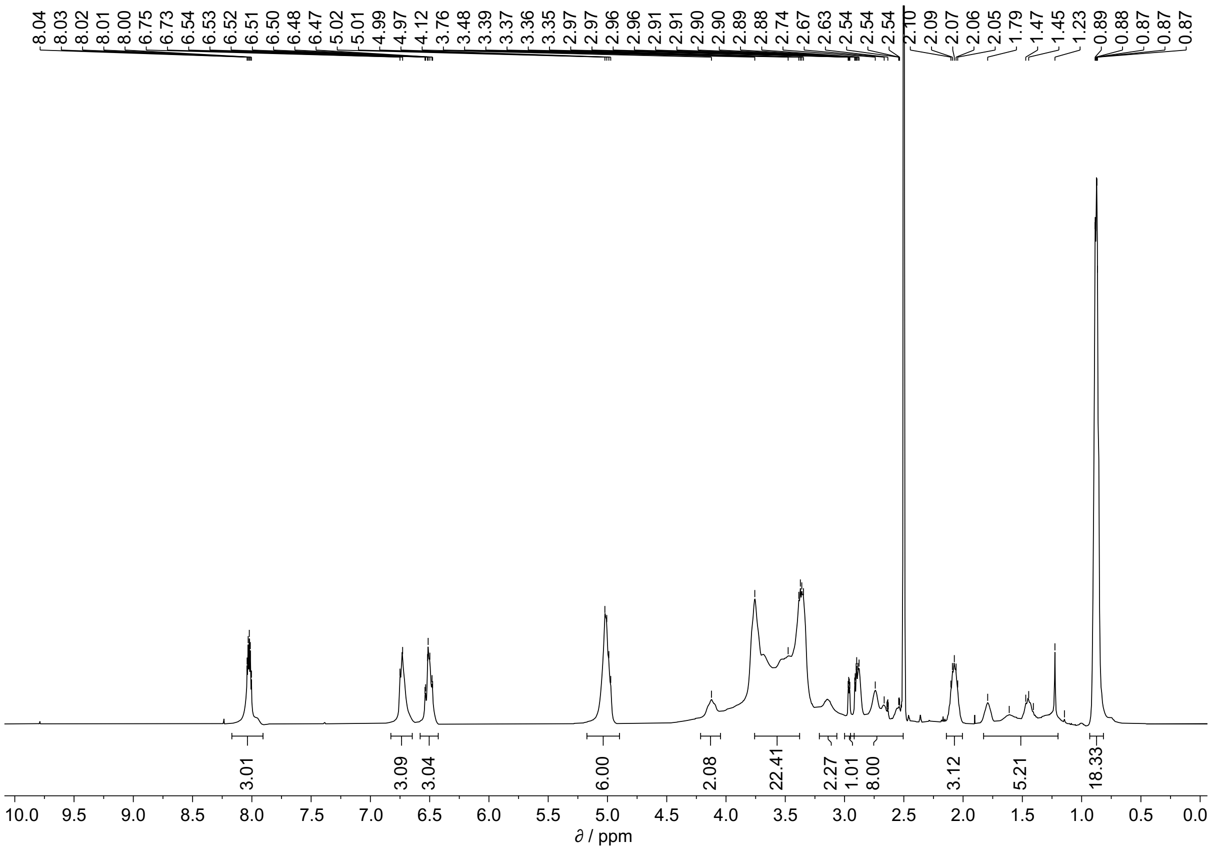

Figure S2: ^{1}H NMR spectrum (500 MHz, DMSO-d_6, 298 K) of **1**.

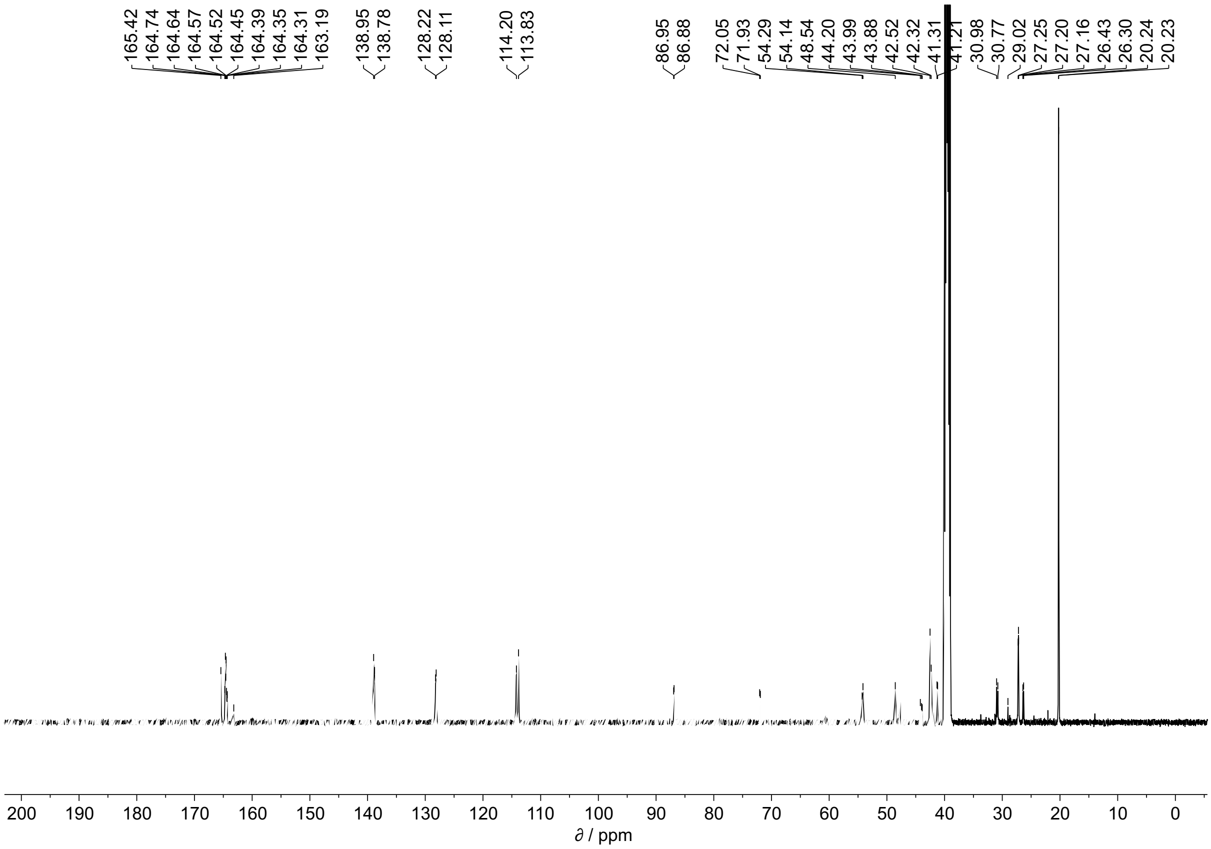

Figure S3: ^{13}C NMR spectrum (126 MHz, DMSO-d_6, 298 K) of **1**.

Synthesis of 2

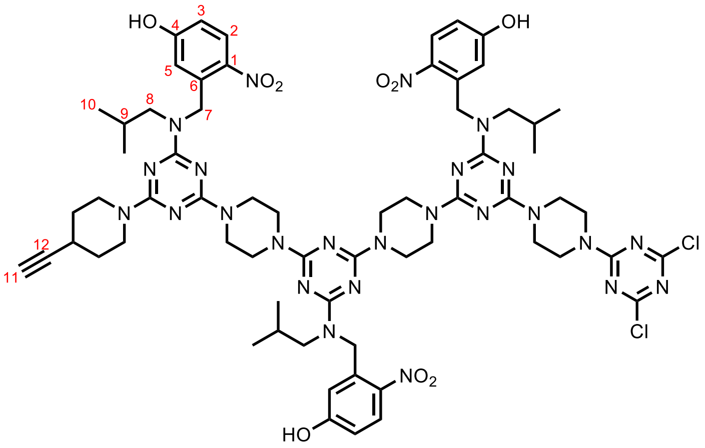

To a solution of **1** (11.8 mg, 0.00932 mmol, 1.0 eq.) in THF (0.5 mL), cyanuric chloride (1.72 mg, 0.00932 mmol, 1.0 eq.) and DIPEA (6.5 µL, 0.037 mmol, 4.0 eq.) were added and the solution was stirred at -78 °C for 1 hour. The reaction was diluted with EtOAc (10 mL) and washed with water (10 mL). The aqueous phase was extracted with EtOAc (3 x 10 mL) and the combined organic phases dried with magnesium sulfate. The solvent was evaporated *in vacuo***.** The residues obtained were purified by silica prep. TLC (DCM/ methanol (97:3)). **2** was obtained as a white foam (6.70 mg, 0.00474 mmol, 51%).

^{1}H NMR (700 MHz, $CDCl_3$): δ_H = 8.07 (br, 3H, H_2), 6.59 (br, 6H, $H_{3,5}$), 5.11 (br, 6H, H_7), 4.37 – 3.16 (br, 34H, H_8, protons of piperazine and piperidine rings), 2.65 and 2.55 (br, 1H, H_{11}, rotamers), 2.11 (br, 3H, H_9), 1.92 – 1.43 (br, 5H, protons of piperidine ring), 0.92 (br, 18H, H_{10}).

^{13}C NMR (176 MHz, $CDCl_3$): δ_C = 170.3, 170.2, 166.2, 166.0, 165.2, 164.1, 164.0 (carbons of triazine rings), 161.3 (C_4), 141.0 ($C_{1/6}$), 139.5 ($C_{1/6}$), 128.4 (C_2), 114.7 ($C_{3/5}$), 113.7 ($C_{3/5}$), 86.8 (C_{12}), 69.6 and 69.5 (C_{11}, rotamers), 55.1 and 55.0 (C_8), 49.2 and 49.1 (C_7), 44.3, 44.2, 43.2, 42.9, 42.8, 42.0 (carbons of piperazine and piperidine rings), 31.4 and 31.2 (piperidine carbon, rotamers), 27.8 (C_9), 27.3 and 27.2 (piperidine carbon, rotamers), 25.0 and 24.9 (piperidine carbon, rotamers), 20.8 (C_{10}).

HRMS (ES+): Calculated for $C_{61}H_{81}N_{22}O_9^+$, 1412.5942; found 1412.5971.

FT-IR (ATR): ν_{max} 3295, 2956, 2926, 2857, 1577, 1530, 1479, 1434, 1367, 1331, 1301, 1259, 1236, 1207, 1158, 994, 844, 807.

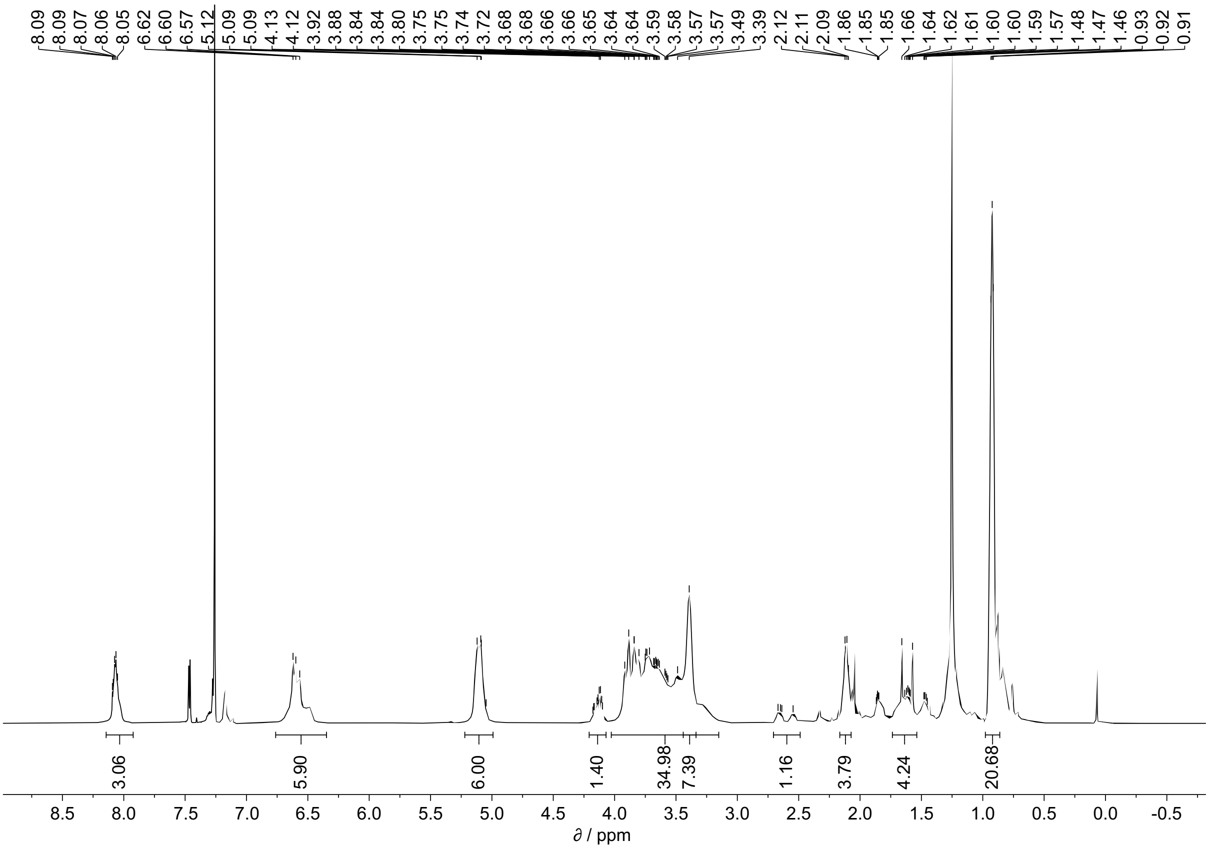

Figure S4: ^{1}H NMR spectrum (700 MHz, $CDCl_3$, 298 K) of **2**.

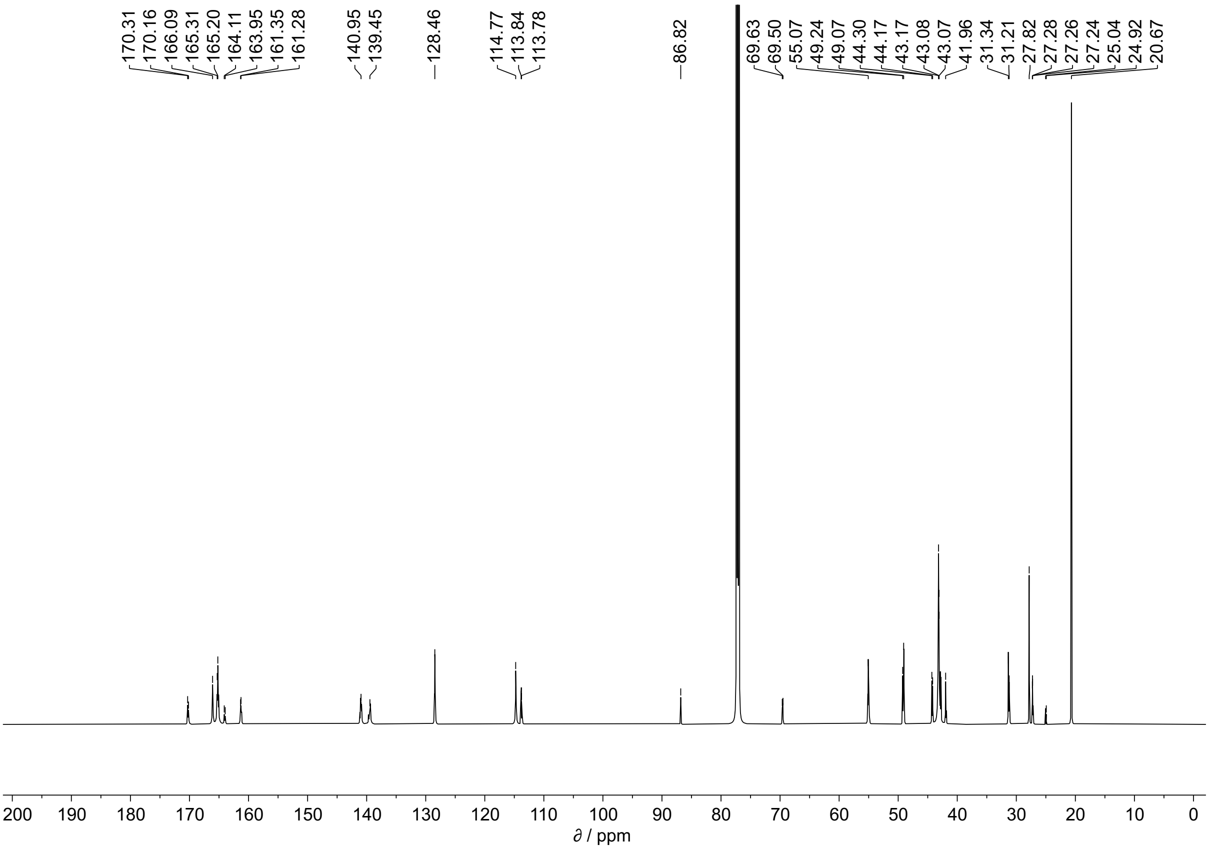

Figure S5: ^{13}C NMR spectrum (176 MHz, $CDCl_3$, 298 K) of **2**.

Synthesis of 5

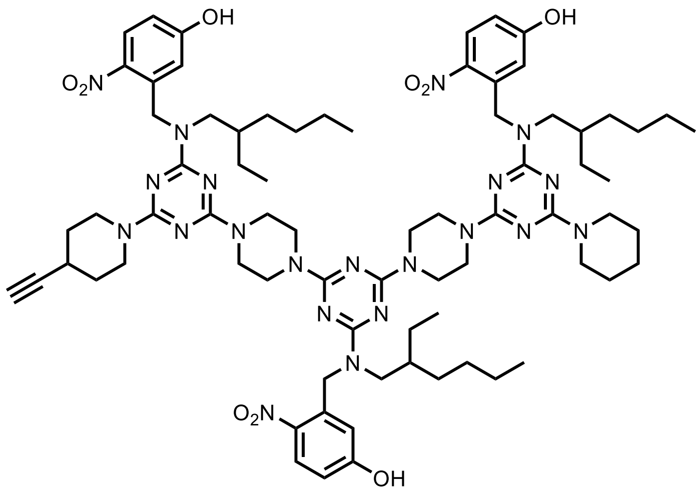

Compound **5** was prepared according to the literature procedure. [5]

Synthesis of 6

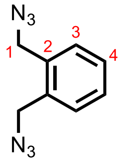

A solution of 1,2-bi(bromomethyl)benzene (0.2967 g, 1.12 mmol, 1.0 eq.) and sodium azide (0.3021 g, 0.464 mmol, 4.0 eq.) in DMF (6 mL) and the resulting mixture was heated at 80°C under reflux for 6 hours. Water (25 mL) was added to the solution which was then extracted by EtOAc (3 × 10 mL). The mixture was washed with a solution of NH4Cl (3 × 15 mL). The combined organic phases were dried ($MgSO_4$), the solvent was evaporated *in vacuo* and the product (0.197 g, 1.05 mmol, 86%) was afforded as a pale-yellow oil.

^{1}H NMR (400 MHz, $CDCl_3$, 298K): δ 7.39 (4H, m, H_3 and H_4), 4.44 (4H, s, H_1)

^{13}C NMR (100 MHz, $CDCl_3$, 298K): δ 134.0 (C_2), 130.2 ($C_{3/4}$), 129.1 ($C_{3/4}$), 52.3 (C_1)

FT-IR (ATR): ν_{max} (cm^{-1}): 2938, 2086, 1492, 1455, 1345, 1289, 1245, 1179, 968, 948, 916, 882, 841, 753, 677, 648, 597, 560, 492, 448

Spectroscopic data matches the literature. [6]

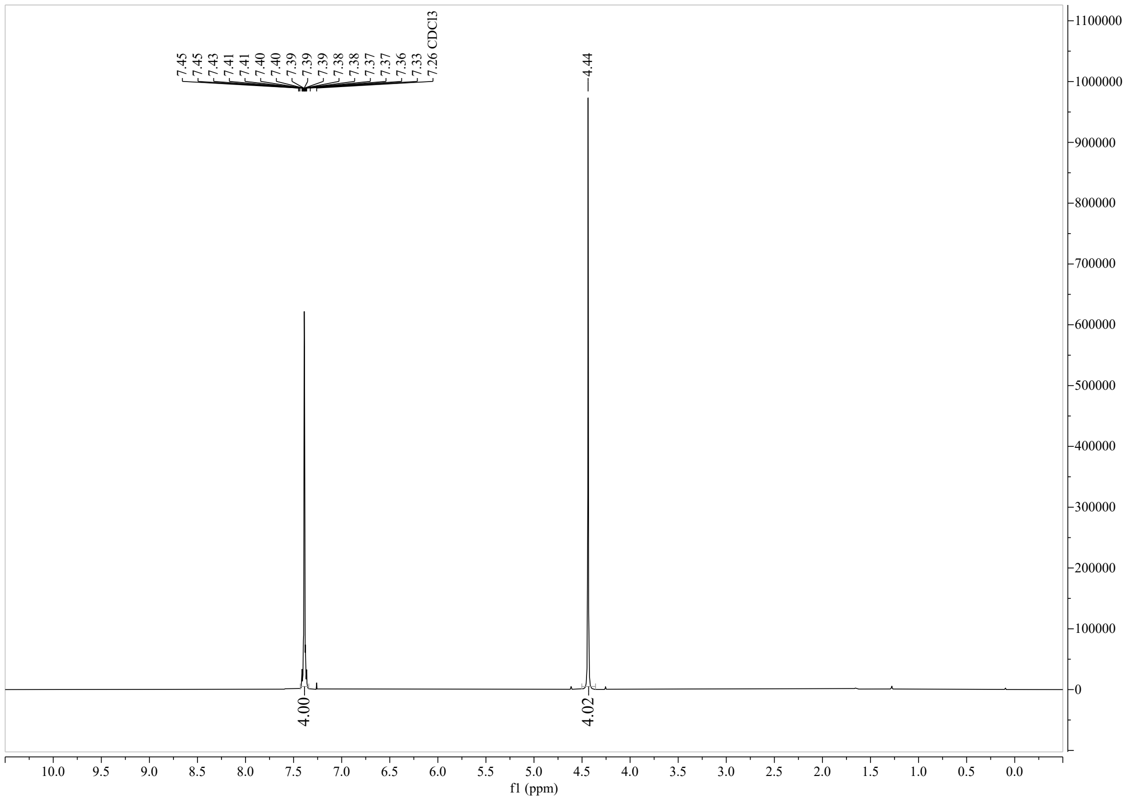

Figure S6: ^{1}H NMR spectrum (400 MHz, $CDCl_3$, 298K) of **6**.

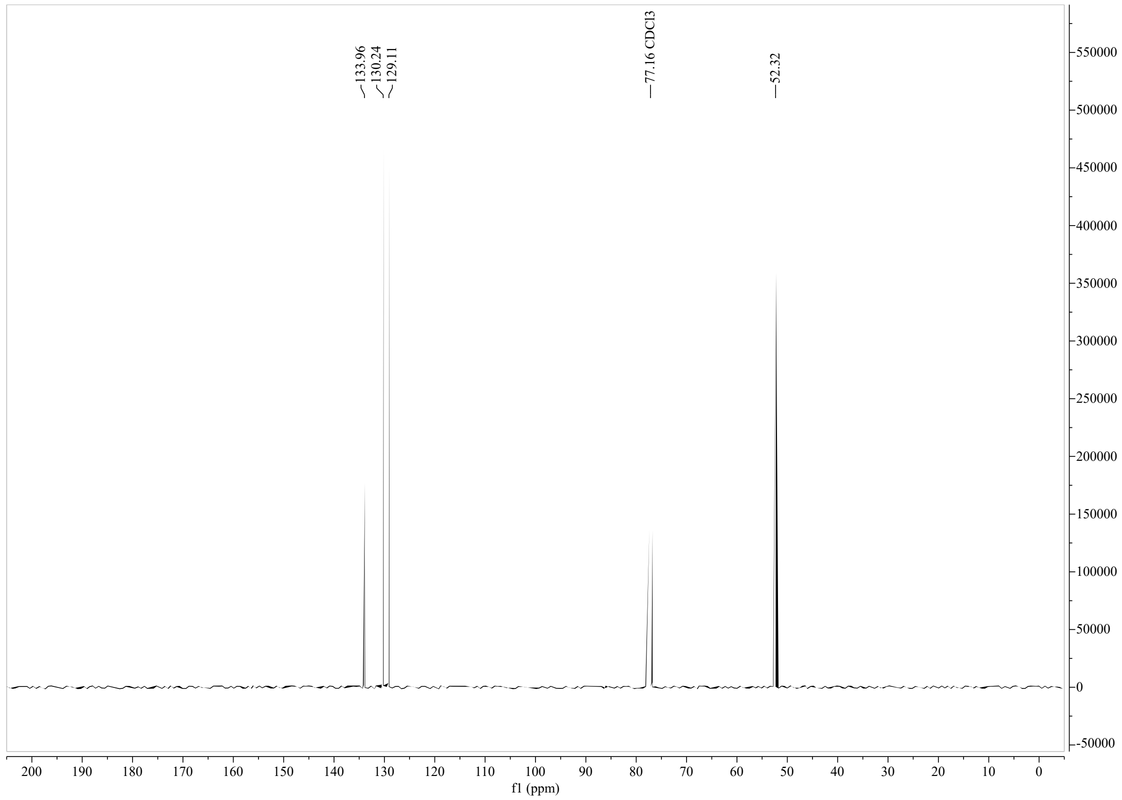

Figure S7: ^{13}C NMR spectrum (100 MHz, $CDCl_3$, 298K) of **6**.

Synthesis of 9

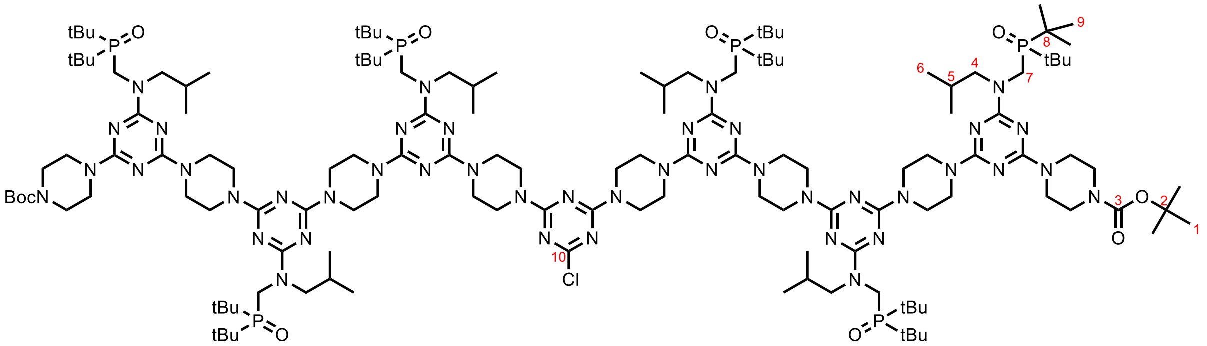

To a solution of cyanuric chloride (3.27 mg, 0.0177 mmol, 1.0 eq.) in dry THF (0.5 mL), **S7** (50.0 mg, 0.0354 mmol, 2.0 eq.) and DIPEA (25 µL, 0.142 mmol, 8.0 eq.) were added and the reaction stirred at room temperature for 4 hours. The reaction was diluted with EtOAc (10 mL) and washed with water (10 mL). The aqueous phase was extracted with EtOAc (3 x 10 mL) and the combined organic phases dried with magnesium sulfate. The solvent was evaporated *in vacuo***.** The residues obtained were purified by prep. TLC (DCM/ methanol (20:1)). **9** was obtained as a white foam (46.1 mg, 0.0157 mmol, 89%).

^{1}H NMR (500 MHz, CDCl$_3$): δ_H = 4.40 (br s, 12H, H$_7$), 3.90 – 3.69 (br, 68H, H$_4$, piperazine protons), 3.44 (br s, 8H, piperazine protons), 2.21 (br, 6H, H$_5$), 1.48 (s, 18H, H$_1$), 1.29 (2 doublets, $^{3}J_{HP}$ = 12.8 Hz, 108H, H$_9$), 0.91 (br s, 36H, H$_6$).

^{13}C NMR (126 MHz, CDCl$_3$): δ_C = 169.8 (C$_{10}$), 165.5, 165.3, 165.1, 164.7 (carbons of triazine rings), 155.0 (C$_3$), 80.1 (C$_2$), 53.2 (C$_4$), 43.6, 43.2, 42.9 (carbons of piperazine rings), 38.1 (d, $^{1}J_{CP}$ = 58.4 Hz, C$_7$), 35.9 (d, $^{1}J_{CP}$ = 55.5 Hz, C$_8$), 28.6 (C$_1$), 26.6 (C$_{5,9}$), 20.7 (C$_6$).

^{31}P NMR (203 MHz, CDCl$_3$): δ_P = 59.50, 59.37.

HRMS (ES+): Calculated for $C_{141}H_{257}ClN_{43}O_{10}P_6^+$, 2933.9032; found 2933.9169.

FT-IR (ATR): ν_{max} 2954, 2923, 2854, 1698, 1528, 1479, 1430, 1366, 1290, 1253, 1206, 1167, 1144, 998, 833, 806, 734, 648, 504, 450.

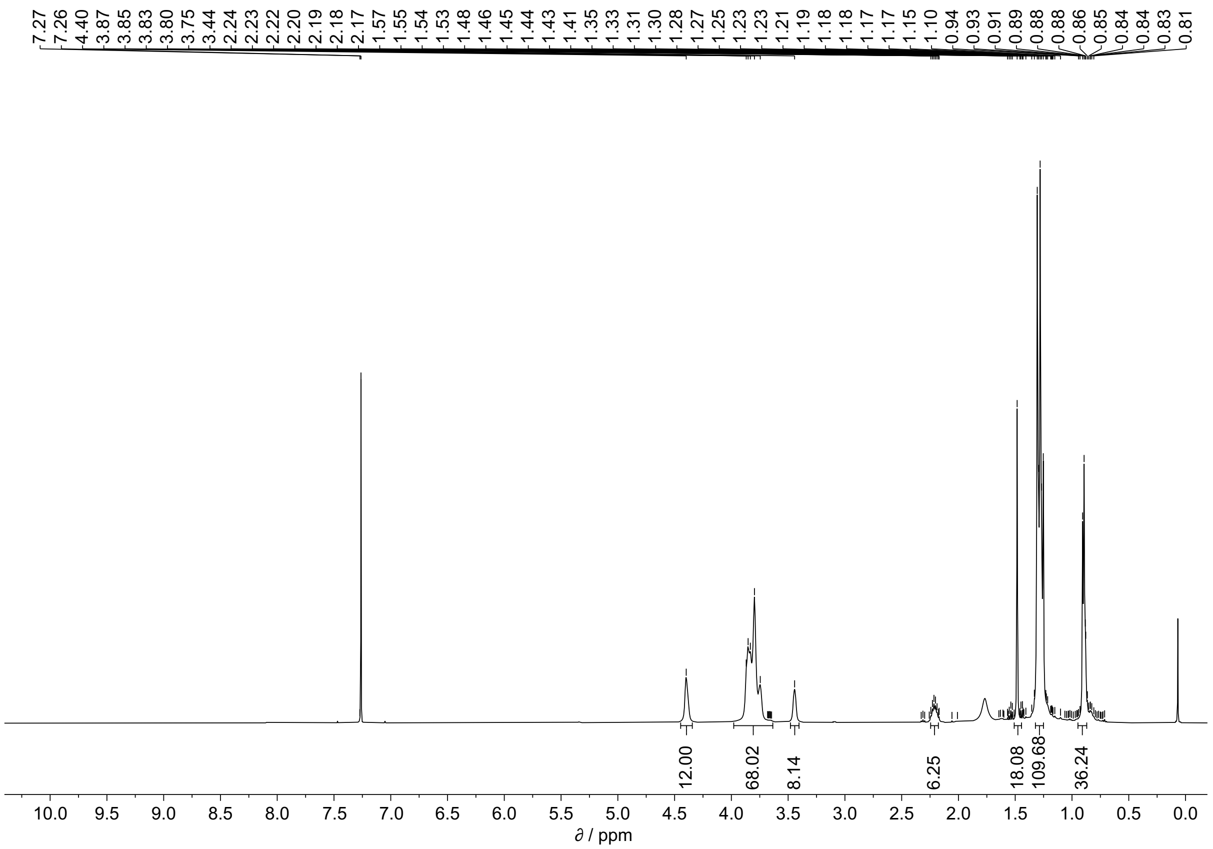

Figure S8: ^{1}H NMR spectrum (500 MHz, $CDCl_3$, 298 K) of **9**.

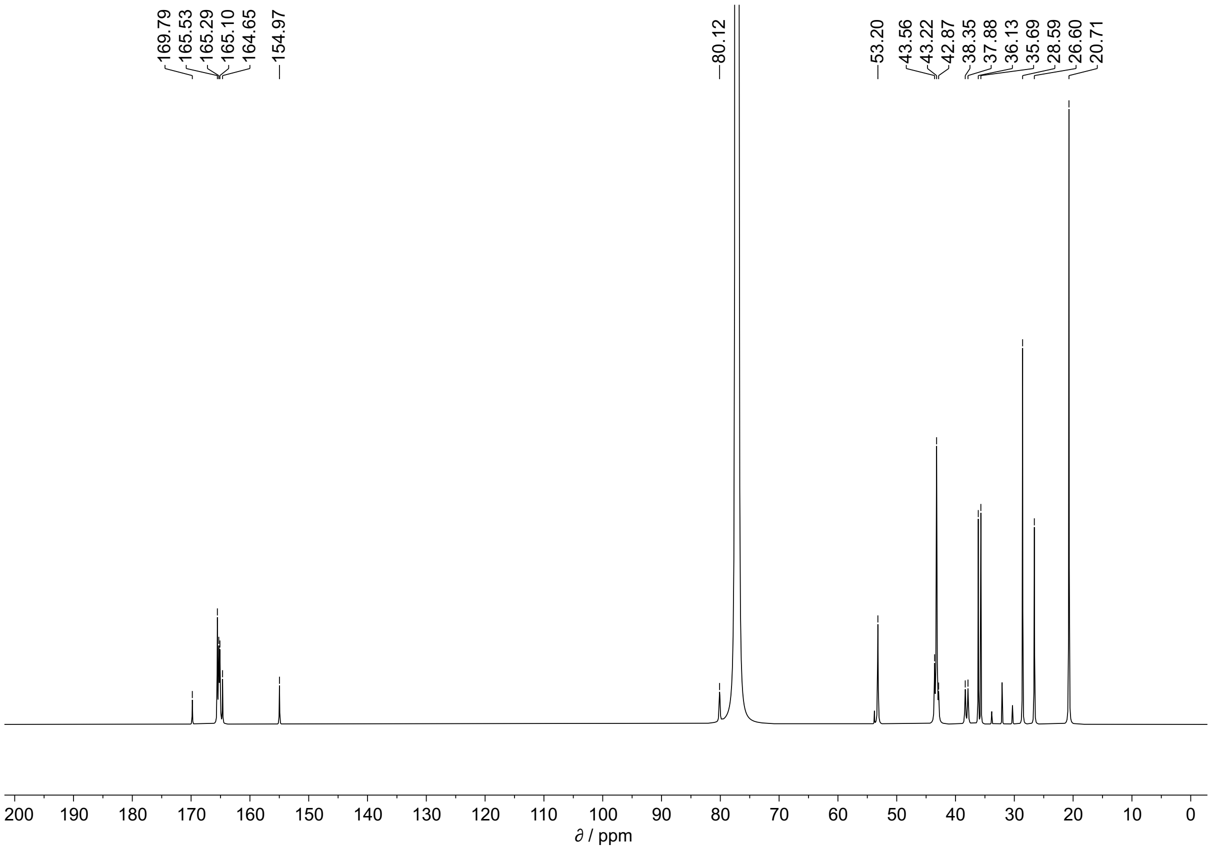

Figure S9: ^{13}C NMR spectrum (126 MHz, $CDCl_3$, 298 K) of **9**.

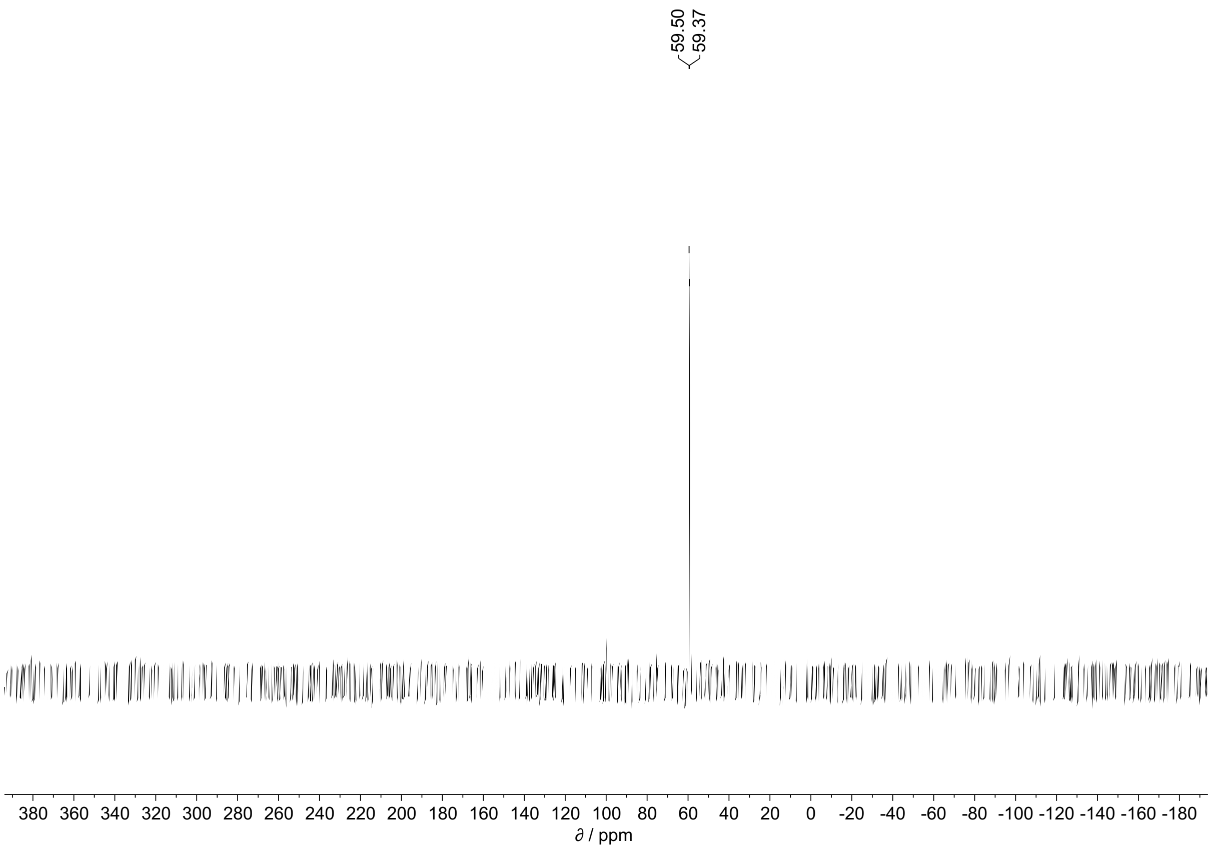

Figure S10: ^{31}P NMR spectrum (203 MHz, $CDCl_3$, 298 K) of **9**.

Synthesis of 10

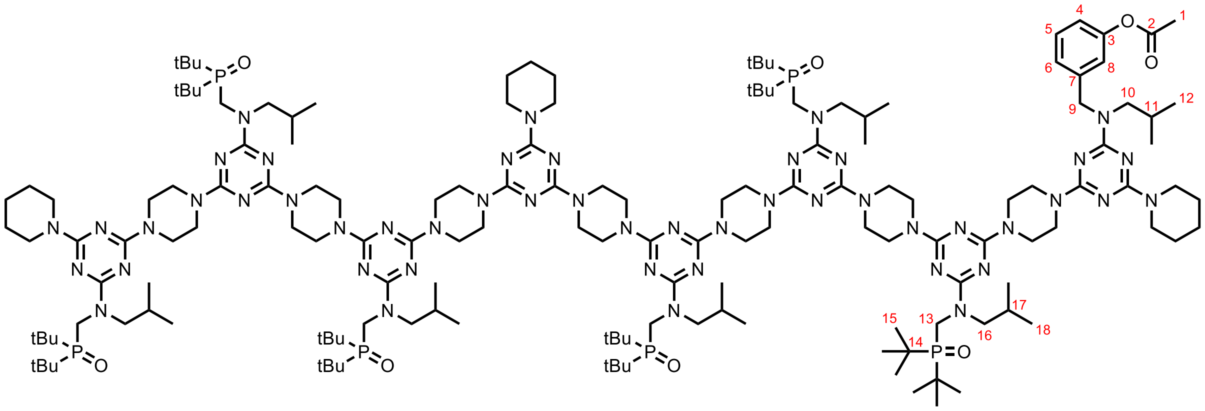

DMF solutions of piperazine (0.7 M), **S1** (0.125 M), **S4** (0.125 M) and DIPEA (0.5 M) were prepared for coupling.

For the Fmoc deprotection: The deprotection was performed using the Liberty Blue Automated Synthesiser. Loaded TentaGel Wang resin **S5** (300 mg, 0.030 mmol) was agitated in a solution of piperazine in DMF (7 mL, 0.7 M, 2 x 10 min). The deprotection solution was then drained and the resin washed with DMF (4 x 5 mL).

The coupling of six dichlorotriazine building blocks was performed using Automated Solid-Phase Synthesis. For each coupling cycle: resin-bound oligomer was agitated in a solution of dichlorotriazine (**S1** or **S4**) (0.125 M, 10 eq.) and DIPEA (0.5 M, 10 eq.) in DMF (5 mL) for 10 mins at 90 °C. The first coupling solution was drained, and the resin washed with DMF (4 x 5 mL). Resin-bound oligomer was then agitated in a solution of piperazine (5 mL, 0.7 M) in DMF for 10 minutes at 90 °C. The second coupling solution was drained, and the resin washed with DMF (4 x 5 mL).

The resin-bound oligomer was swollen in NMP before being stirred in a solution of piperidine (0.05 mL, 0.507 μmol) and DIPEA (0.22 mL, 1.27 μmol) in NMP (5 mL) for 1 hour at 90 °C. The coupling solution was drained, and the resin was washed with DMF (4 × 5 mL).

To cleave the oligomer from the resin: TFA (4.5 mL), TIS (0.25 mL) and DCM (0.25 mL) were added to the resin and the mixture was agitated for 2 hours. The resin was washed with DCM (5 x 5 mL), the filtrates were combined, and solvent was removed in *vacuo*. The obtained residue was purified by flash column chromatography (SiO_2, C18, 30-100% THF in water) to yield **pDAAAPAAAp** (27.3 mg, 8.75 μmol, 29% based on initial resin loading).

pDAAAPAAAp (27.3 mg, 8.75 μmol), EDC.HCl (24.7 mg, 0.129 mmol, 15 eq.) and DMAP (16.7 mg, 0.139 mmol) were suspended in dry THF (2 mL). Glacial AcOH (7.4 uL, 0.13 mmol, 15 eq,) was added, and the reaction stirred at room temperature overnight. The mixture was concentrated, dissolved in ethyl acetate (2 mL) and washed with HCl (2.0 mL, 1.0 M). The organic layer was dried (anhydrous $MgSO_4$) and solvent removed *in vacuo*. Crude product was purified by flash column chromatography (SiO_2, C18, 30-100 % THF in water) to yield **10** (21.3 mg, 6.73 μmol, 77%).

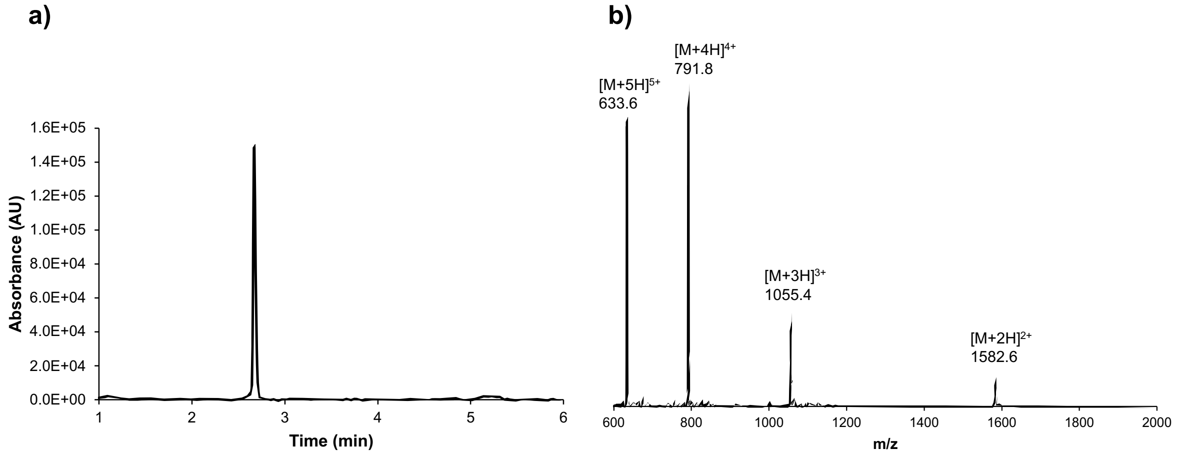

Figure S11: a) UPLC trace of **pAAAPAAAD*p, b)** ESI-MS of **pAAAPAAAD*p**. Calculated masses: 1582.6 $[M+2H]^{2+}$, 1055.0 $[M+3H]^{3+}$, 791.3 $[M+4H]^{4+}$, 633.0 $[M+5H]^{5+}$; Mass found (ESI$^+$): 1582.6 $[M+2H]^{2+}$, 1055.4 $[M+3H]^{3+}$, 791.8 $[M+4H]^{4+}$, 633.6 $[M+5H]^{5+}$. UPLC conditions: C_4 column at 40 °C using a 30-100% gradient of THF/formic acid (0.1%) in water/formic acid (0.1%) over 4 minutes, then 100% THF/formic acid (0.1%) over 2 minutes.

^{1}H NMR (400 MHz, $CDCl_3$, 298K): 7.28 (1H, m, H_5 overlaps with solvent), 7.11 (1H, d, J = 7.6 Hz, H_4), 6.95 (2H, m, H_6 and H_8), 4.83 (2H, m, H_9), 4.40 (12H, s, H_{13}), 3.96-3.71 (80H, br m, H_{16}, piperazine and piperidine on carbons next to nitrogen), 3.33 (2H, m, H_{10}), 2.27 (3H, s, H_1), 2.21 (6H, m, H_{17}), 2.07 (1H, m, H_{11}), 1.64-1.54 (18H, m, piperidine), 1.29 (108H, d, J = 12.9 Hz, H_{15}), 0.90 (42H, m, H_{12} and H_{18})

^{31}P NMR (162 MHz, $CDCl_3$), δ 58.82, 58.76, 58.64

HRMS (ES$^+$): Calculated for $C_{158}H_{279}N_{48}O_8P_6^+$, 3164.1405; found 3164.1357

IR $\tilde{\nu}$ (cm^{-1}): 2952, 1690, 1527, 1479, 1430, 1367, 1255, 1205, 1146, 997, 807, 730

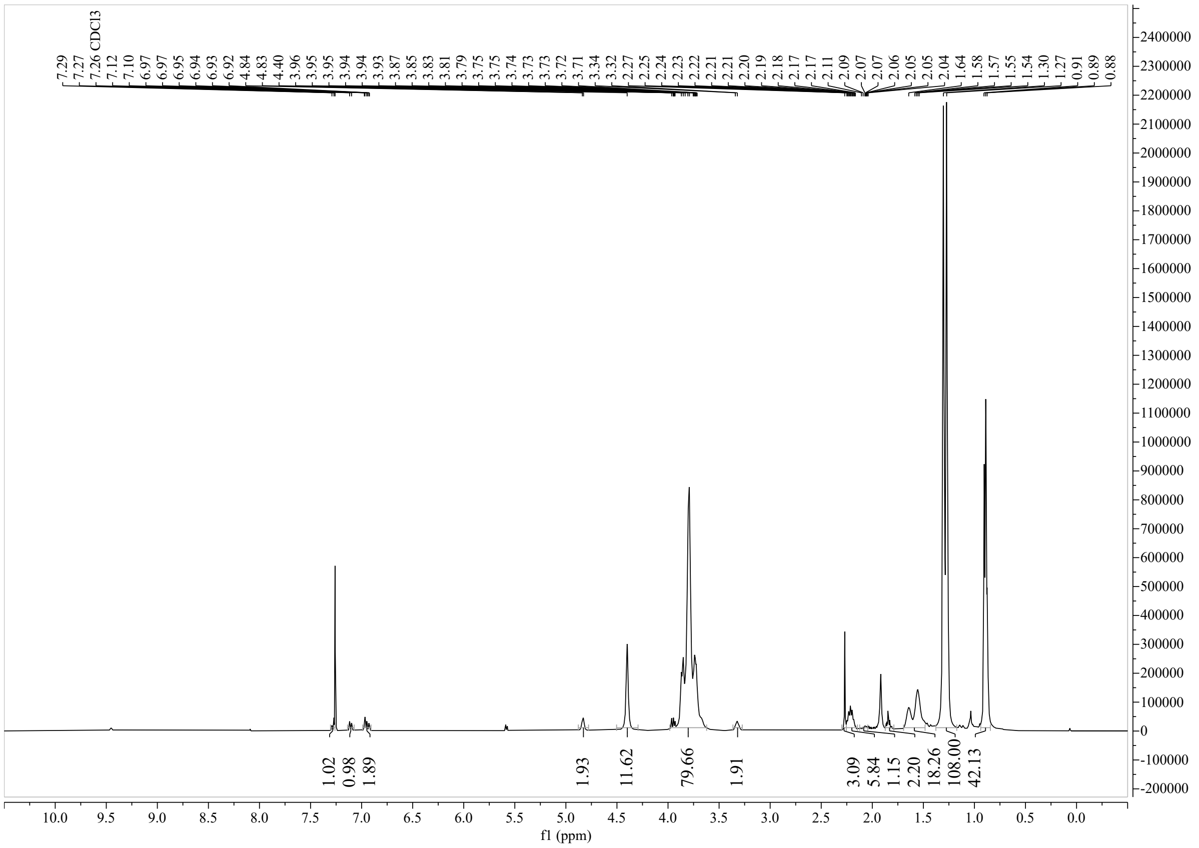

Figure S12: ^{1}H NMR spectrum (400 MHz, $CDCl_3$, 298K) of **10**.

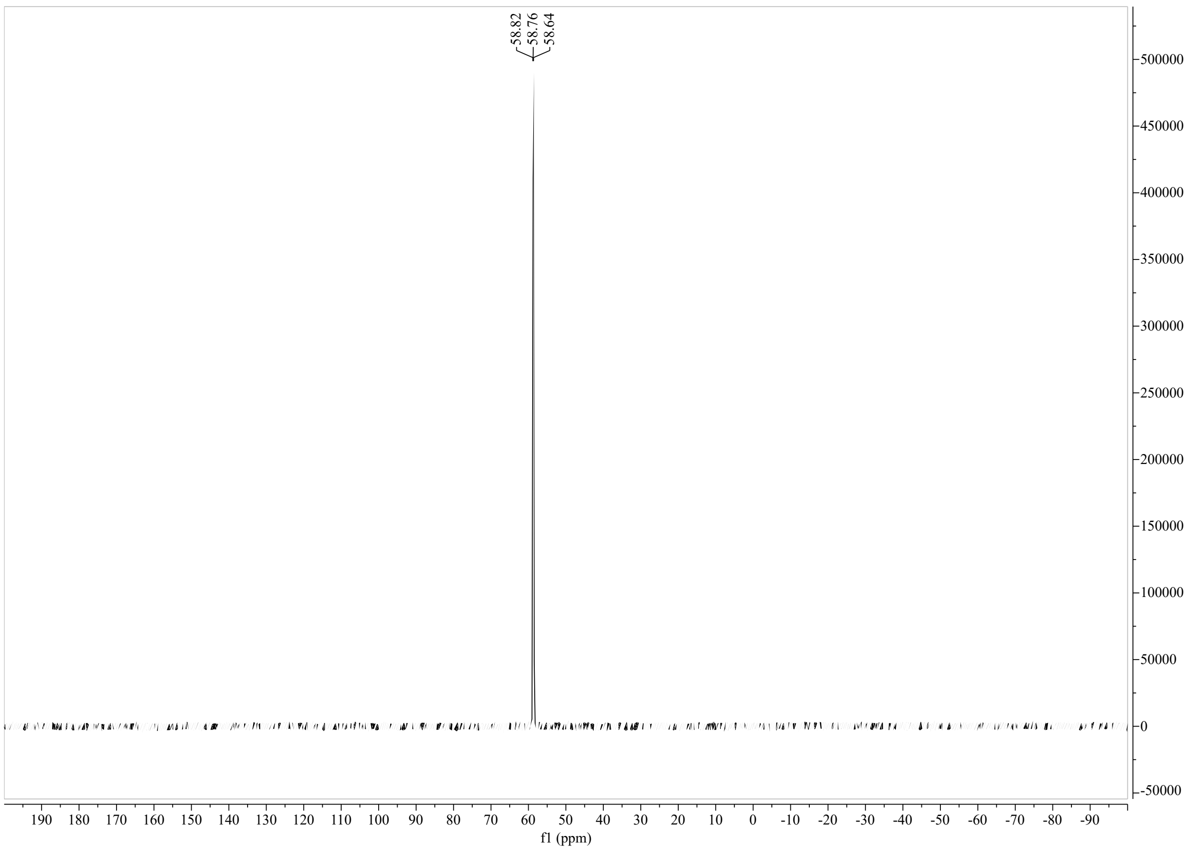

Figure S13: ^{31}P NMR spectrum (162 MHz, $CDCl_3$, 298K) of **10**.

Synthesis of 11

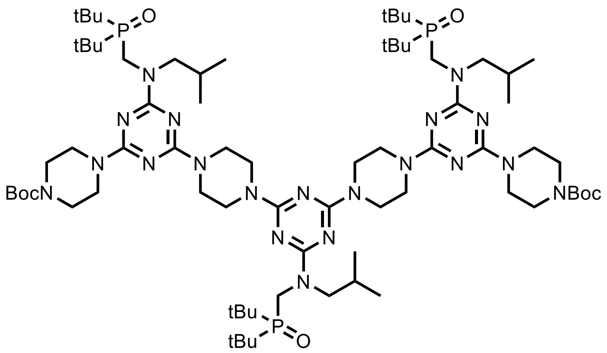

Compound **11** was synthesised according to the literature procedure. [7]

Synthesis of 12

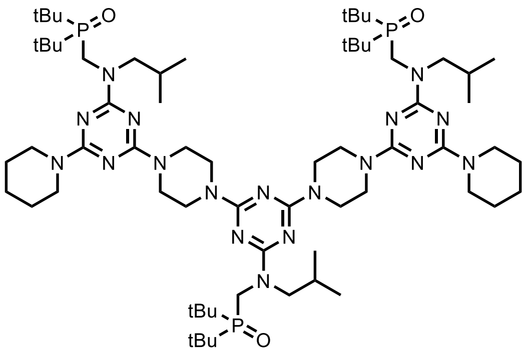

Compound **12** was synthesised as previously described.[1]

3. Procedures for Templating Studies

Ligation with S_NAr

Stock solutions of **2**, **9**, **11** and *N*-Boc-piperazine were made up in dichloromethane. Stock solutions of **1** were made up in mixtures with DIPEA such that the DIPEA was at ten times the concentration of the **1**. Calculated amounts of the stocks of relevant species of **9**, **11**, *N*-Boc-piperazine and the **1** and DIPEA stock were mixed and diluted with a calculated volume of dichloromethane. To start the reactions, a calculated amount of **2** was added and the reaction vials were shaken vigorously. Reactions were left at room temperature for either 2 hours (templated) or 1 day (untemplated). Aliquots of the reaction mixture (15 μL) were taken and diluted in THF (135 μL) and injected into the UPLC instrument (2 μL).

Ligation with CuAAC

Stock solutions of **5**, **6**, **10, 12** and Cu(I) TBTA were made up in dichloromethane. Calculated amounts of the stocks of relevant species of **5**, **6**, **10** and **12** were added to 1 ml reaction vials and dried under a flow of nitrogen. A calculated amount of Cu(I) TBTA was added to each vial and the reaction volume made up to 200 μL in dichloromethane. Reactions were stirred at room temperature for 48 hours. They were then quenched by evaporation of solvent under a flow of nitrogen, 200 μL DMSO was added and the vials stirred at room temperature for 2 hours. Aliquots of the reaction mixture (10 μL) were taken and diluted in MeCN (100 μL) and injected into the UPLC instrument (2 μL).

References

1. Troselj, P., Bolgar, P., Ballester, P. & Hunter, C. A. High-Fidelity Sequence-Selective Duplex Formation by Recognition-Encoded Melamine Oligomers. *J. Am. Chem. Soc.* **143**, 8669–8678 (2021).
2. Balduzzi, F. *et al.* Length and Sequence-Selective Polymer Synthesis Templated by a Combination of Covalent and Noncovalent Base-Pairing Interactions. *J. Am. Chem. Soc.* **146**, 32837–32847 (2024).
3. Dhiman, M. *et al.* Selective Duplex Formation in Mixed Sequence Libraries of Synthetic Polymers. *J. Am. Chem. Soc.* **146**, 9326–9334 (2024).
4. Dhiman, M., Cabot, R. & Hunter, C. A. Efficient automated solid-phase synthesis of recognition-encoded melamine oligomers. *Chem. Sci.* (2024) doi:10.1039/D4SC00973H.
5. Smith, J. T., Baixeras Buye, J., Iddon, B., Soloviev, D. O. & Hunter, C. A. Template-Directed Synthesis of Recognition-Encoded Melamine Oligomers Using a Base-Filling Strategy. *J. Am. Chem. Soc.* **147**, 18284–18294 (2025).
6. Yoshiyama, H., Shibata, N., Sato, T., Nakamura, S. & Toru, T. Synthesis of trifluoroethoxy-coated binuclear phthalocyanines with click spacers and investigation of their clamshell behaviour. *Org. Biomol. Chem.* **7**, 2265–2269 (2009).
7. Dhiman, M., Smith, J. T. & Hunter, C. A. Supramolecular assembly properties of a mixed-sequence recognition-encoded melamine oligomer. *Org. Biomol. Chem.* **23**, 6948–6956 (2025).
